# Finding common ground: Toward comparable indicators of adaptive capacity of tree species to a changing climate

**DOI:** 10.1002/ece3.8024

**Published:** 2021-09-02

**Authors:** Samuel Royer‐Tardif, Laura Boisvert‐Marsh, Julie Godbout, Nathalie Isabel, Isabelle Aubin

**Affiliations:** ^1^ Natural Resources Canada Canadian Forest Service Great Lakes Forestry Centre Sault Sainte Marie ON Canada; ^2^ Centre d'enseignement et de recherche en foresterie de Sainte‐Foy inc. (CERFO) Québec QC Canada; ^3^ Ministère des Forêts de la Faune et des Parcs du Québec Direction de la recherche forestière Québec QC Canada; ^4^ Natural Resources Canada Canadian Forest Service Laurentian Forestry Centre Québec QC Canada; ^5^ Centre for Forest Research Université du Québec à Montréal Montréal QC Canada

**Keywords:** data comparability, functional traits, genetic differentiation, genetic diversity, genetic exchange, interspecific hybridization, phenotypic plasticity

## Abstract

Adaptive capacity, one of the three determinants of vulnerability to climate change, is defined as the capacity of species to persist in their current location by coping with novel environmental conditions through acclimation and/or evolution. Although studies have identified indicators of adaptive capacity, few have assessed this capacity in a quantitative way that is comparable across tree species. Yet, such multispecies assessments are needed by forest management and conservation programs to refine vulnerability assessments and to guide the choice of adaptation measures. In this paper, we propose a framework to quantitatively evaluate five key components of tree adaptive capacity to climate change: individual adaptation through phenotypic plasticity, population phenotypic diversity as influenced by genetic diversity, genetic exchange within populations, genetic exchange between populations, and genetic exchange between species. For each component, we define the main mechanisms that underlie adaptive capacity and present associated metrics that can be used as indices. To illustrate the use of this framework, we evaluate the relative adaptive capacity of 26 northeastern North American tree species using values reported in the literature. Our results show adaptive capacity to be highly variable among species and between components of adaptive capacity, such that no one species ranks consistently across all components. On average, the conifer *Picea glauca* and the broadleaves *Acer rubrum* and *A. saccharinum* show the greatest adaptive capacity among the 26 species we documented, whereas the conifers *Picea rubens* and *Thuja occidentalis*, and the broadleaf *Ostrya virginiana* possess the lowest. We discuss limitations that arise when comparing adaptive capacity among species, including poor data availability and comparability issues in metrics derived from different methods or studies. The breadth of data required for such an assessment exemplifies the multidisciplinary nature of adaptive capacity and the necessity of continued cross‐collaboration to better anticipate the impacts of a changing climate.

## Graphical abstract

Adaptive capacity has yet to be assessed in a quantitative way that is comparable across tree species, precluding its widespread use in assessments of vulnerability to climate change. We propose a framework to quantitatively evaluate five key components of adaptive capacity: individual adaptation through phenotypic plasticity, population phenotypic diversity as influenced by genetic diversity, genetic exchange within populations, genetic exchange between populations, and genetic exchange between species. We discuss limitations and roadblocks of our approach, including inconsistency in data availability across species and comparability issues for metrics derived from different methods or studies.

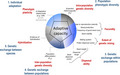

## 1 INTRODUCTION

The unprecedented rate of climate change is expected to expose tree populations to climatic conditions beyond those to which they are adapted, possibly jeopardizing forest health and survival (McKenney et al., 2011; Thuiller et al., 2005). Trees represent the foundation of forest habitats, play an important role in regulating the global carbon cycle, and sustain resource‐based economies. Anticipating their potential response to climate change is therefore of primary importance in forest management and conservation (Belote et al., 2017; Seidl et al., 2018). Understanding which species might be favored or threatened by climate change is necessary to guide species selection for reforestation and conservation programs (Aitken & Bemmels, 2016; Chmura et al., 2011) and to prioritize management actions aimed at promoting resistance, resilience, or transition of forest ecosystems (Bolte et al., 2009; Millar et al., 2007; Nagel et al., 2017).

In response to climate change, tree species may either persist in their current location, migrate to “track” their climatic niche, or be extirpated (Aitken et al., 2008). However, many studies suggest that changes in tree climatic niches over the next century will exceed the migration capacity of tree species (Aubin et al., 2018; Boisvert‐Marsh et al., 2014; Dobrowski et al., 2013; Dyderski et al., 2018; Serra‐Diaz et al., 2014). To evaluate the ability of species to persist in place, much research in recent years has focused on assessing ecological vulnerability to climate change (Aubin et al., 2018; Belote et al., 2018; Foden et al., 2019; Seidl et al., 2018; Wade et al., 2017). Three elements define species’ vulnerability to climate change: (a) exposure, the magnitude of projected environmental change; (b) sensitivity, the degree to which a species is likely to be negatively impacted by this change; and (c) adaptive capacity, the capacity of species to cope with and adapt to novel conditions (Glick et al., 2011). For some authors, migration capacity is part of species’ adaptive capacity to environmental change (e.g., de los Ríos et al., 2018; Thurman et al., 2020). However, for sessile organisms such as trees, migration capacity involves a different suite of traits than those required to persist in place (see Boisvert‐Marsh et al., 2020 for a framework to evaluate the migration capacity of tree species). In this paper, we focus on adaptive capacity as the capacity of tree species to face and adapt to changing climatic conditions in their current location.

So far, vulnerability assessments have been based mainly on exposure, with recent inclusion of species’ sensitivity made possible because ecological data in the appropriate format are becoming more widely available (Aubin et al., 2016; de los Ríos et al., 2018). However, adaptive capacity has rarely been integrated into such assessments and remains the least known determinant of vulnerability (de los Ríos et al., 2018). Yet, recent findings show the importance of adaptive capacity in modulating the response of ecological systems to climate change (Benito Garzón et al., 2019; Bouchard et al., 2019). As such, adaptive capacity should be considered in projections of species’ future climatic niches (Peterson et al., 2019) as well as in forest management and conservation planning (Walsworth et al., 2019). Hence, there is a pressing need to characterize tree adaptive capacity to better anticipate species’ ability to persist in their current location over the coming decades (Aitken et al., 2008; Alfaro et al., 2014; Nicotra et al., 2015; Price et al., 2013; Scotti, 2010).

Adaptive capacity is a function of a wide range of biological, physiological, ecological, and evolutionary processes that act at various spatial and temporal scales, from phenotypic plasticity of individual genotypes (Nicotra et al., 2010) to population genetic diversity (Jump et al., 2009) as well as species evolutionary potential as determined by life‐history traits (Alfaro et al., 2014), and intra‐ (Savolainen et al., 2007) and interspecific genetic exchanges (Menon et al., 2020). Assessing species capacity to adapt as a whole is thus an interdisciplinary exercise that lies at the intersection of ecophysiology, population genetics, community ecology, and biological conservation. Additionally, because variability is a core concept of adaptive capacity, a large quantity of data is needed to evaluate its components (e.g., phenotypic plasticity Violle et al., 2012).

Advances in genomics and phenotyping methods over the last decade offer opportunities to evaluate adaptive capacity at an unprecedented resolution, even for less‐studied species. For example, the advent of next‐generation sequencing and new genotyping methods (Andrews et al., 2016) has paved the way for new research fields such as landscape genomics (e.g., Borrell et al., 2020; Hall & Beissinger, 2014; Rellstab et al., 2015). Combined with the invaluable knowledge acquired through common garden experiments and provenance trials (e.g., Depardieu et al., 2020; Morgenstern & Teich, 1969; Rehfeldt et al., 1999), we can now evaluate tree genotype–environment and genotype–phenotype relationships and better understand the selective pressures that come into play (Depardieu et al., 2021; Housset et al., 2018; Nadeau et al., 2015). Such “comprehensive” approaches to adaptive capacity can identify adaptive mismatches in tree populations to future (or even current) climatic conditions (e.g., Borrell et al., 2020; Ingvarsson & Bernhardsson, 2020; St Clair & Howe, 2007).

Conversely, from a forest management perspective, a multispecies approach is needed to compare species’ relative ability to persist under a changing climate and to inform adaptation measures and conservation efforts. However, scaling comprehensive evaluations of adaptive capacity across species remains a considerable challenge. Such evaluations are resource intensive to conduct, so they remain limited to only a few well studied and economically important tree species (e.g., *Pseudotsuga menziesii*; St Clair & Howe, 2007, *Pinus contorta var. latifolia*; Rehfeldt et al., 2018, Mahony et al., 2020) or species of special concern (e.g., *Betula nana*; Borrell et al., 2020). As a result, the few multispecies comparative assessments of adaptive capacity that we are aware of are based on well characterized but coarse qualitative metrics indicative of adaptive capacity (e.g., number of populations in Wade et al., 2016, regeneration capacity in Potter et al., 2017). They usually exclude key indicators of adaptive capacity such as population genetic diversity (e.g., Butt & Gallagher, 2018) or rely on their surrogates (e.g., seed zone number, pollination vector, and disjunct populations as surrogates of genetic diversity in Potter et al., 2017).

Another barrier to comparative assessments of adaptive capacity is the presence of comparability issues in physiological and genomic data between tree species. Many aspects of forest science were developed from an autecological perspective, that is, by studying one species at a time, while methods and technologies have evolved over time. In some cases, it may be difficult to compare results based on earlier methods with ones obtained using more recent methods. Considering the limitations of past comparative efforts, a transdisciplinary framework is needed to evaluate adaptive capacity in a way that is both comparable across species while better integrating quantitative metrics that encompass phenotypic plasticity and evolutionary potential.

This synthesis aims to provide assistance to modelers and practitioners in selecting metrics to characterize species’ adaptive capacity. We build on the current state of knowledge on tree species biology to develop a new transdisciplinary and comparative framework for tree adaptive capacity. Using a set of 26 abundant tree species in northeastern North America, we document and synthesize available data into five distinct classes of quantitative indicators that correspond to the five components of adaptive capacity. We discuss the quality of this information and evaluate its comparability across tree species. Finally, we take this opportunity to identify knowledge gaps and roadblocks, explore solutions, and outline future research needs toward the development of robust indicators of adaptive capacity.

## 2 METHODS

### 2.1 Documenting the five components of adaptive capacity

**FIGURE 1:**
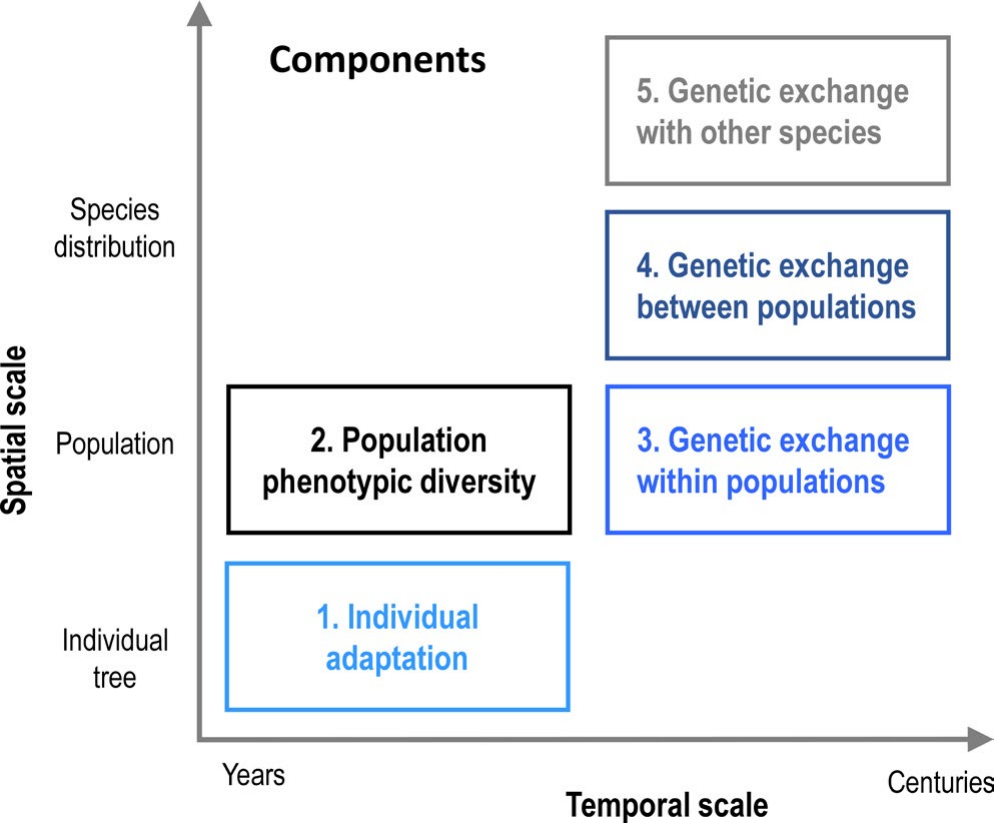
Five components of adaptive capacity, as presented along spatio‐temporal axes. At the scale of individuals, trees can adapt to changing conditions through phenotypic plasticity: the capacity of a single genotype (i.e., a unique set of genes) to express different phenotypes under different environmental conditions. At larger spatial scales, the diversity of phenotypes within a given population may contain individuals that are already adapted. In addition, the rate of genetic exchange among individuals within a population—as determined by tree fecundity, mating system, and pollen and seed dispersal ability—may accelerate evolutionary adaptation. At broader spatial scales, the level of genetic differentiation between populations might be indicative of localized adaptations and informs the likelihood of movement between, and possibly induce evolutionary adaptation, in vulnerable populations. As well, evolutionary adaptation could be facilitated by genetic exchanges between two or more closely related species (hybridization/introgression) that occur naturally in contact zones. Along the temporal axis, phenotypic plasticity and diversity are expected to provide adaptation in the short term (i.e., within an individual's lifetime, generally a few decades but up to several centuries). Conversely, evolutionary processes associated with genetic exchanges within and between populations/species typically occur over multiple generations. See Appendix S1 for more details

**FIGURE 2:**
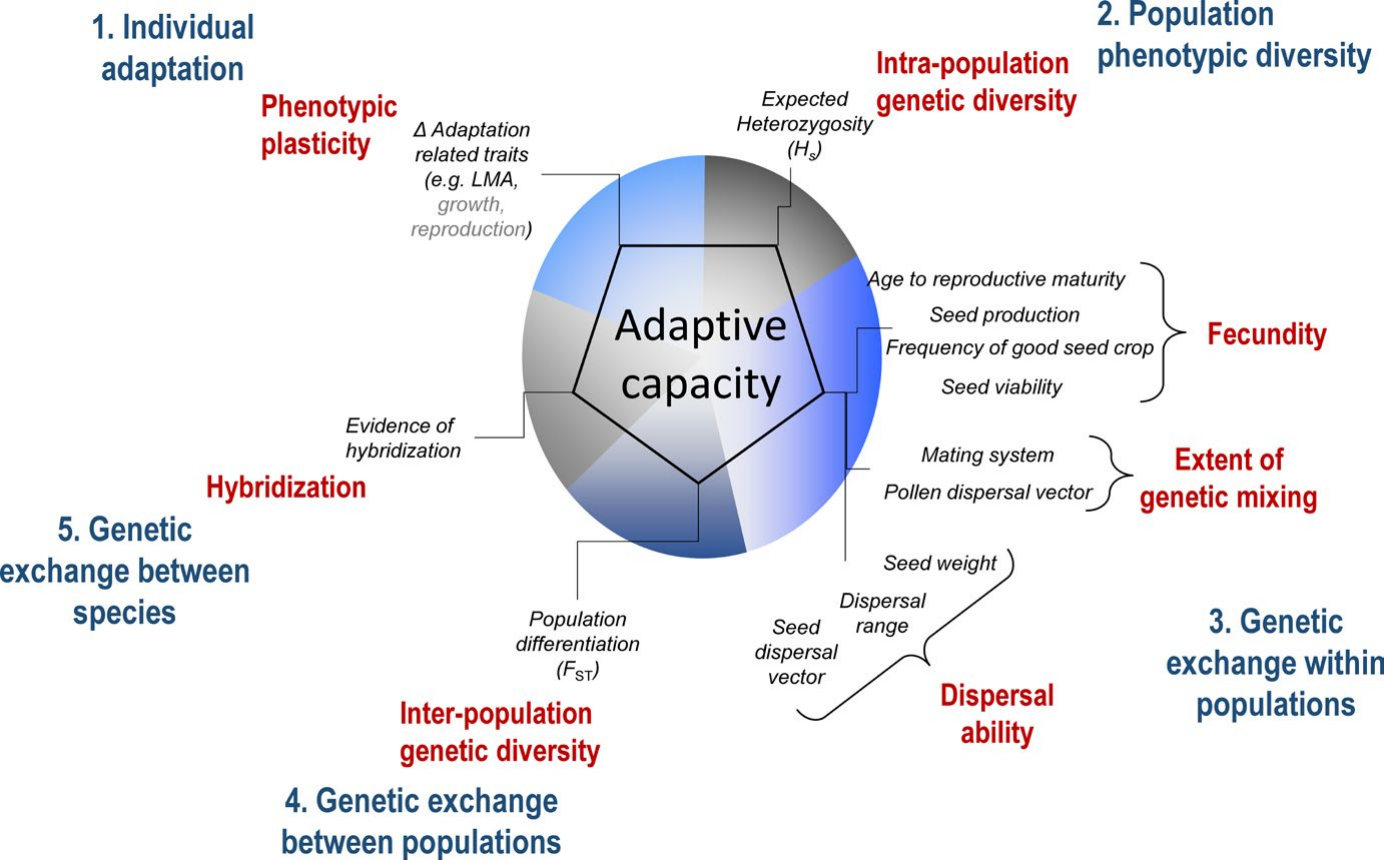
Framework used to characterize tree species‐specific adaptive capacity. The five adaptation components are presented in dark blue while red refers to mechanisms. The metrics used to quantify each mechanism are presented in black. The colors of the circle in the center correspond to colors used in Figure 1. Adaptation‐related traits in gray (growth and reproduction) represent important traits that were not considered in this study because they were not sufficiently documented (see Appendix S2: Table A2.1 for details). See Figure 1 for component description and Appendices S1–S5 for details on metric selection and index development

For each of the five components of tree adaptive capacity (Figure 1), we identified the main mechanisms (Table 1, further detailed in Appendix S1) and associated metrics (see Figure 2) that underlie differences in species’ adaptive capacity. We selected metrics that were available and comparable across as many species as possible, and independent of the experimental context in which they were measured. For these reasons, we were often limited to data acquired using conventional techniques instead of the most recent. To illustrate our framework and identify possible roadblocks and limitations, we (a) documented the selected metrics for each component of adaptive capacity for 26 tree species dominant in northeastern North America (see list in Appendix S2: Table A2.1) and (b) conducted a meta‐analysis on the metrics’ inter‐ and intraspecific variability to assess their potential to characterize differences in species adaptive capacity.

**TABLE 1:** Description of the main tree species' mechanisms underlying each component of adaptive capacity

Component	Mechanism	Description	Relationship with adaptive capacity	References
1. Individual adaptation	Phenotypic plasticity	Ability of a single tree to persist in altered environmental conditions via phenotypic plasticity, that is, by adjusting its morphology or physiology to new conditions.	↗ Increases the range of conditions (e.g., altered climate) under which a single individual can survive, grow, or reproduce.	Nicotra et al. (2010), Valladares et al. (2014)
2. Population phenotypic diversity	Intrapopulation genetic diversity	Gene diversity within a population.	↗ Increases the chances that some genotypes will be adapted to future conditions and provides material required for future evolutionary potential.	Jump et al. (2009), Hoffmann and Sgrò (2011)
3. Genetic exchange within populations	Fecundity	Number of seeds produced per time period (in this case, 40 years) as influenced by age to sexual maturity and viable seed production once mature.	↗ Increases the probability of yielding better adapted individuals. Rate of evolution increases as generation time decreases.	Franks et al. (2014)
	Extent of genetic mixing	Probability that seeds originate from unrelated genetic materials (i.e., outcrossing rate).	↗ Increases effective population size, the probability of yielding better adapted individuals, and the rate of evolution. Also reduces potential for inbreeding and its negative evolutionary consequences.	Charlesworth and Charlesworth (1987), Franks et al. (2014), Alfaro et al. (2014)
	Dispersal ability	Distance which seeds are dispersed.	↗ Increases the area where seeds could potentially fall, and hence the probability of finding a suitable habitat for seedling development. Also increases gene flow within and between populations over the long term.	Vittoz and Engler (2007)
4. Genetic exchange between populations	Interpopulation genetic diversity	Genetic differentiation between populations	↗ Increases potential of translocation or breeding of adapted individuals	Aitken and Bemmels (2016)
5. Genetic exchange between species	Hybridization	Genetic exchange between interfertile species	↗ Increases probability of yielding individuals better suited to an altered climate.	Arnold and Kunte (2017), Suarez‐Gonzalez, Lexer, et al. (2018)

#### 2.1.1 Component #1: Individual adaptation through phenotypic plasticity

Nicotra et al. (2010) listed key plant traits (e.g., leaf mass per unit area, stomatal size, flowering time, and seed number) for which phenotypic plasticity could potentially confer adaptation to climate change. To evaluate the availability of suitable data to measure phenotypic plasticity, we searched the Web of Science database for studies that explored plasticity in these traits for our set of 26 tree species (see Appendix S2 for details and results of the queries). Among the six traits identified as suitable, three were sufficiently documented to compare phenotypic plasticity for our 26 species. These traits are leaf mass per area (LMA), timing of budburst, and seed production, with data for 81%, 65%, and 65% of the species documented, respectively (see Table A2.1 in Appendix S2). Stomata size and density, flowering time, and timing of bud set are not sufficiently documented to be considered here.

To illustrate interspecific variation and differences in phenotypic plasticity, we used plasticity of LMA to different levels of light availability. LMA was chosen because it determines species’ fitness within its environment (Poorter et al., 2009), affects many ecosystem processes (Poorter et al., 2009; Wright et al., 2004), and is one of the most commonly measured plant traits (Kattge et al., 2020). Similarly, light availability is an important environmental factor because it influences the balance between photosynthesis efficiency, heat stress, and water deficit, and has often been explored in comparative studies of temperate trees (Goulet & Bellefleur, 1986; Lei & Lechowicz, 1998; Paquette et al., 2012; Sanford et al., 2003). We chose the Environmentally Standardized Plasticity Index (ESPI; Valladares et al., 2006) as the plasticity metric for this component. This index corresponds to the difference in LMA between low and high light environments divided by the difference in light availability (% full sunlight, log‐transformed) between each of the environments (see Appendix S2 for details). We also used the standard error in ESPI indices as a measure of our level of confidence in our estimates of species phenotypic plasticity.

#### 2.1.2 Component #2: Population phenotypic diversity as influenced by genetic diversity

Different metrics can be used to capture tree genetic diversity (Appendix S1). Here, we used the level of expected heterozygosity within tree populations (denoted as *H*_s_; Figure 2). This metric corresponds to the allelic richness weighted by the frequency of each allele in a given population (see Appendix S1 for more details). Expected heterozygosity is purely statistical and is not influenced by nonrandom mating or other factors that can affect the observed proportion of heterozygotes (Freeman & Herron, 2014). Therefore, *H*_s_ is a good estimator of genetic diversity that is comparable across species experiencing different conditions that influence demography. To obtain a comparable measure of genetic diversity across species, we conducted a survey of published *H*_s_ estimates for the 26 tree species listed in Table A2.1 (Appendix S2).

When *H*_s_ was provided for multiple loci or multiple populations, we computed the mean of the values to yield a single estimate per marker type per study per species. Since expected heterozygosity can be calculated at different scales (e.g., individual, population, regional, or range‐wide), we limited our data collection to values obtained at the population level (sampling locations separated by at least 100 km). For more details, see Appendix S3.

Markers were classified into the following types: allozyme, haplotypes, RAPD, RFLP, SNP, cpDNA SSR, mtDNA SSR, and nuclear SSR (see Appendix S1 for details about each marker type). A separate class was ascribed to each SSR marker type because of the strong difference in *H*_s_ values between mtDNA, cpDNA, and nuclear SSR. To explore the influence of the different marker types and tree species on *H*_s_ values, we applied a linear fixed‐effects model using two factors—tree species (23 levels) and marker type (8 levels). Pairwise contrasts between each marker type and allozymes were further evaluated by testing the significance of model parameters for each level of the factor “marker” using the summary.lm() function in the R software (v.3.6.3., R Core Team, 2017). A Bonferroni‐corrected level of significance was used to account for multiple hypothesis testing.

To overcome the variation introduced by the different marker types, we restricted our classification of population genetic diversity to *H*_s_ estimates from allozymes, RAPD, RFLP, and SNP. These values represented the bulk of our *H*_s_ values (67 of 93 values) and were more consistent across studies. We also calculated the standard error in species‐level estimates to evaluate the confidence in our estimates of genetic diversity.

#### 2.1.3 Component #3: Genetic exchange within populations as a function of life‐history traits

Life‐history traits selected to capture tree capacity for genetic exchange are presented in Figure 2 (see Appendix S1 for more details). We quantified the amount of genetic exchange within populations as the number of viable seeds produced that are genetically distinct from their parents (thereafter called “number of viable seeds that are genetically distinct” or NVSGD). This index describes the genetic relatedness of seeds produced as determined by the probability of fertilization via outcrossing (i.e., mating system) and traits influencing gene flow (pollination vector). We used these last two traits as adjustment factors for tree fecundity, which is calculated following Boisvert‐Marsh et al. (2020), that is, the number of viable seeds produced per hectare over a period of 40 years. The NVSGD index was further adjusted to account for the seed dispersal ability of each species. We used an index of dispersal ability that combines information on seed weight, seed dispersal vector, and metrics of dispersal distance, as described in Boisvert‐Marsh et al. (2020). The complete details of the NVSGD calculation are provided in Appendix S4: Table A4.1. The final NVSGD values were expressed as the number of viable seeds produced per hectare over a period of 40 years (in millions) adjusted by seed dispersal ability and potential for genetic mixing.

#### 2.1.4 Component #4: Genetic exchange between populations as determined by population differentiation

Different metrics have been developed to evaluate population genetic differentiation, among which the most commonly measured and reported metric is *F*_ST_—a genetic index based on allele frequencies at a locus (Weir & Cockerham, 1984). Analogous to beta‐diversity indices in ecology, *F*_ST_ corresponds to the proportion of a species genetic diversity that is not shared among populations.

To document genetic differentiation for our set of 26 tree species, we performed a survey of *F*_ST_ values reported in the literature (see Appendix S5 for more details). The extent of gene flow between populations is influenced by pollen and seed dispersal and can exert a strong influence on population genetic differentiation. Accordingly, genetic markers that are inherited from a single parent, such as cpDNA and mtDNA, may exhibit different genetic patterns than those inherited from both parents, such as nuclear DNA. To account for this possible bias, we tested for the effect of molecular markers on *F*_ST_ values with a linear fixed‐effects model using two factors—species (20 levels) and marker type (7 levels), distinguishing between markers inherited by both parents (allozymes, RAPD, nuclear SNP, and nuclear SSR) and those inherited from only one parent (maternal cpDNA, maternal mtDNA, and paternal cpDNA). As done for *H*_s_, pairwise contrasts with Bonferroni correction were performed between each marker type and allozymes.

We used allozyme, RAPD, nuclear SNP, nuclear SSR, and paternal cpDNA estimates of population differentiation to rank our species because they provided comparable estimates of genetic differentiation (see the Section 3), had lower within‐species variation, and constituted a considerable proportion of values in our dataset. We calculated standard error in species‐level values to evaluate the confidence in our estimates of population differentiation.

Study sampling effort relative to a species’ range size may be a confounding factor contributing to observed intraspecific variation in the *F*_ST_ values shown here. Specifically, studies that surveyed populations at the regional scale may show less differentiation than studies conducted across the entire geographic range of a species. We tested the potential influence of study scale on *F*_ST_ values by comparing values recorded at a regional scale (i.e., a province or a few states) with those encompassing a species’ entire range. For this test, we used Wilcoxon signed‐rank tests due to the highly skewed distribution of *F*_ST_ residuals.

#### 2.1.5 Component #5: Potential for genetic exchange between species through hybridization

We compiled evidence from the literature of frequent hybridization that can occur naturally or artificially between our 26 species and any other tree species. We distinguished between hybridization occurring between native species in North America from that occurring with exotic species. This distinction is an indication of whether adaptation though hybridization could occur naturally or through human‐assisted translocations (see Appendix S1 for a discussion on the topic).

### 2.2 Index development

Using the selected metrics, we developed five indices corresponding to each of the five components of adaptive capacity. Each index ranks the relative adaptive capacity of our 26 tree species, by assigning them to one of five classes: very low (1), low (2), intermediate (3), high (4), and very high (5). For numeric indices, that is, LMA plasticity to light, intrapopulation genetic diversity, NVSGD, and population differentiation, we used a *k*‐means clustering to partition species into the five classes. We used the k‐means function implemented in the R software with *k* = 5 groups, 10 iterations and 10 random steps. For hybridization capacity, we defined three classes: species unable to form hybrids (low, 2), species forming hybrids with only one species (intermediate, 3), and species forming hybrids with many other species (high, 4). For each species, the classes were averaged across the five components to provide an average index of adaptive capacity.

The classification based on clustering is dependent on the extent of the gradient in adaptive capacity that was captured across our study species. Indeed, even for similar species, this clustering approach could detect differences between defined groups. However, there is evidence that our set of 26 tree species could in fact represents a broad range of adaptive capacity. For example, *Fagus grandifolia* is well known for the plasticity of its leaves, as compared to species in the genus *Populus* (Goulet & Bellefleur, 1986). Likewise, *Picea glauca* and *Pinus resinosa* are species with particularly high (Rajora et al., 2005) and low (Hamrick et al., 1992; Mosseler et al., 1992) levels of population genetic diversity.

### 2.3 Confidence scores

We attributed a confidence score to each index score based on the quality and quantity of the information available. For components 1, 2, and 4, confidence levels were based on the standard error of the numerical values and clustered into four equally sized classes (low, intermediate, high, and very high). The fifth class was attributed to singletons and depicts the lowest level of confidence (very low). For component 3, the confidence scores are based on the availability of trait data for reproductive capacity and dispersal ability mechanisms following Boisvert‐Marsh et al. (2020). For component 5, a high confidence score was attributed for each observation supported by a reference.

## 3 RESULTS

### 3.1 Component #1: Individual adaptation through phenotypic plasticity

**FIGURE 3:**
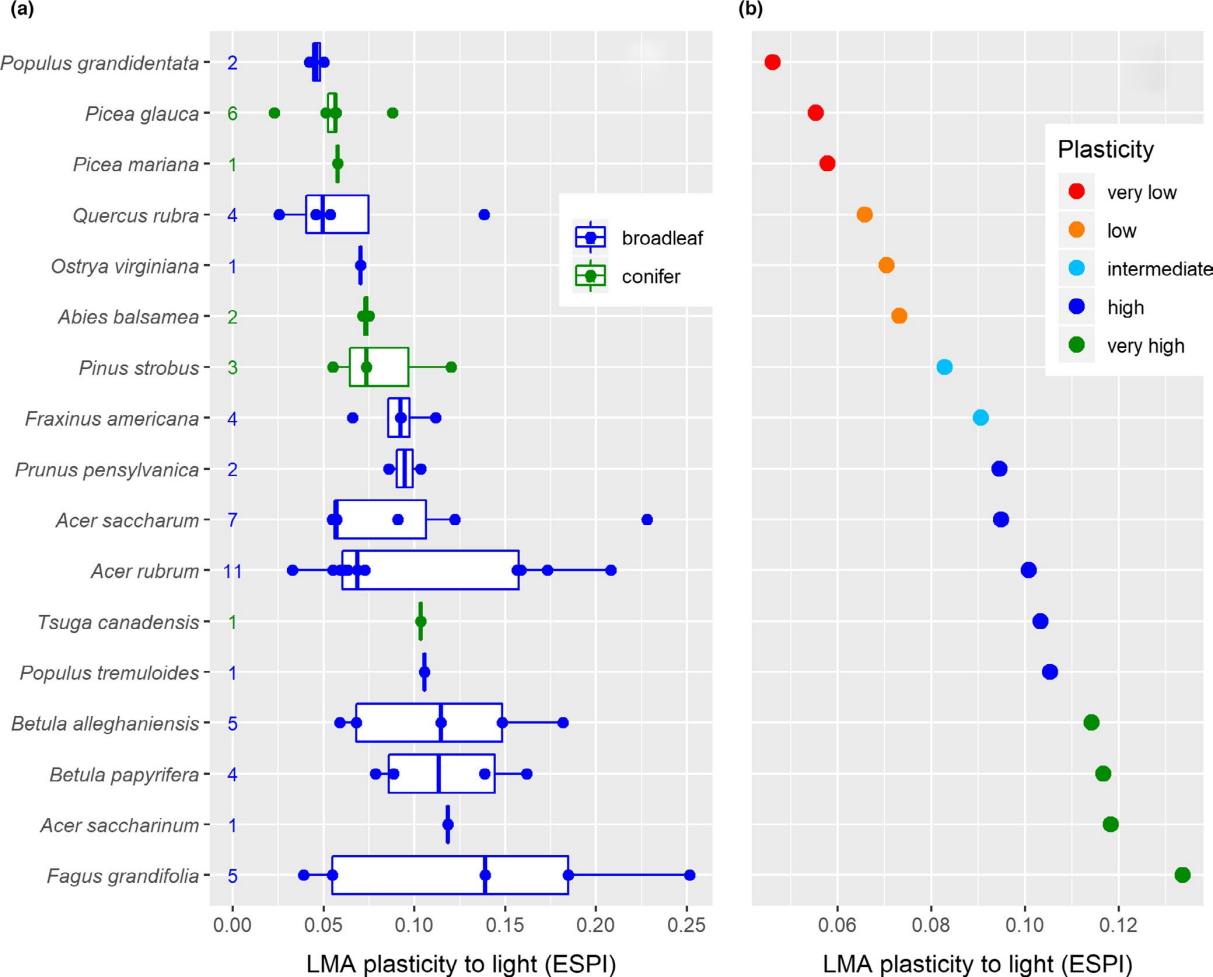
Plasticity of leaf mass per area (LMA) to light availability, calculated as the Environmentally Standardized Plasticity Index (ESPI). 17 of the 26 species were considered in this meta‐analysis while no data were found in the literature for the remaining nine species (i.e., hatched cells in the “LMA plasticity to light” column in Figure 6). ESPI was calculated using the log of light availability (% of full sunlight) to account for the nonlinear relationship of LMA with light. (a) Distribution of the collected plasticity values as well as the number of observations obtained from the literature provided to the left of boxplots. (b) Average plasticity for each species. The five colors represent five groups with increasing levels of plasticity as classified using a *k*‐means clustering. See Appendix S2 for more details on the meta‐analysis

Results in Figure 3 show considerable interspecific variation in LMA plasticity to light, with *Fagus grandifolia* being the most plastic species and *Populus grandidentata* the least plastic. There is also important intraspecific variation in LMA plasticity, notably for *Acer rubrum* and *Fagus grandifolia*. The level of intraspecific variation in these species covers almost the entire range of plasticity measures found in this study.

### 3.2 Component #2: Population phenotypic diversity as influenced by genetic diversity

**FIGURE 4:**
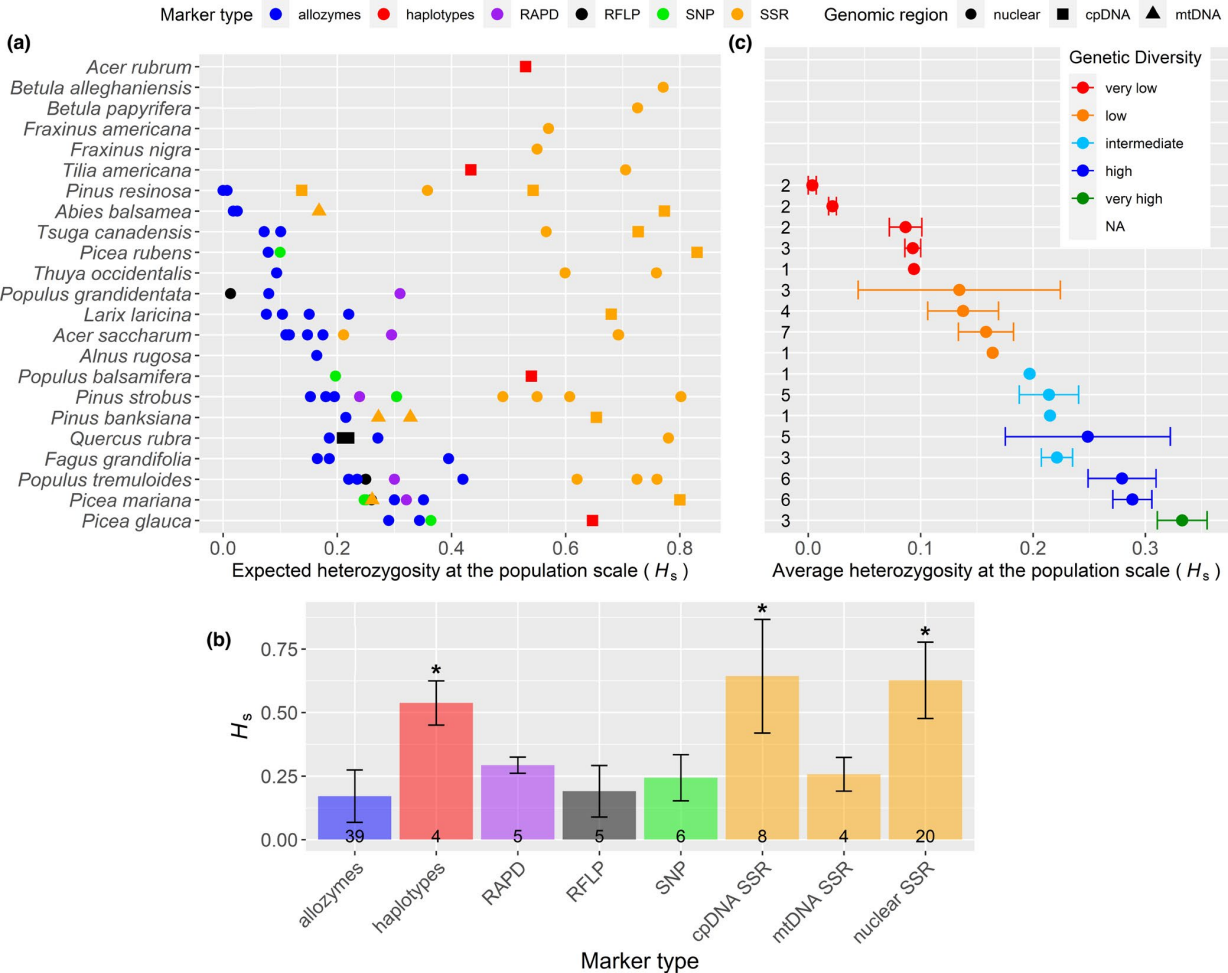
Expected heterozygosity at the population scale (*H*_s_). 23 of the 26 species were considered in this meta‐analysis while no data were found in the literature for the remaining three species (i.e., hatched cells in the “Intrapopulation genetic diversity” column in Figure 6). When multiple populations of a species were surveyed in the same study, we report the average value. (a) Raw *H*_s_ values per species from all studies considered. (b) *H*_s_ averaged across species per marker type (note that SSR markers were separated by genomic region). Asterisks denote significant (*p* < .007) differences from allozyme markers in a linear fixed‐effects model accounting for the effect of species (22 levels) and marker type (8 levels). Error bars correspond to the standard deviation, and the number of replicates is indicated at the bottom of each bar. Haplotypes were all derived from cpDNA, and diversity was provided in the original publications as haplotype diversity (see Appendix S4 for details). (c) Average *H*_s_ per species based on comparable markers, that is, values derived from markers without an asterisk in panel b. Error bars correspond to the standard error. For each species (*y*‐axis), numbers on the left indicate the number of observations. Average *H*_s_ were classified into five classes using a *k*‐means clustering

Population genetic diversity values retrieved from the literature reveal large variation in *H*_s_ estimates between—but also within—the same tree species (Figure 4a). Tree species and marker types both had a highly significant effect on *H*_s_ values, with marker type explaining most of the variation (marker SS = 3.39, species SS = 1.16, residuals SS = 0.61). Haplotypes, cpDNA SSR, and nuclear SSR all yielded significantly higher *H*_s_ values than allozymes (*p* < .001; Figure 4a,b). The data provided in Figure 4 also indicate that allozymes, RAPD, RFLP, and SNPs yield comparable estimates of *H*_s_.

Considering only comparable genetic markers (i.e., *H*_s_ estimates from allozymes, RAPD, RFLP, and SNP) reveals that *Pinus resinosa* has the lowest level of genetic diversity and *Picea glauca* has the highest (Figure 4c). For some species, however, the low number of available *H*_s_ estimates may influence their ranking (e.g., only one *H*_s_ value available for *Alnus incana* subsp. *rugosa*).

### 3.3 Component #3: Genetic exchange within populations as a function of life‐history traits

There was considerable variation in the number of viable seeds that are genetically distinct index (NVSGD; expressed as millions of seeds per hectare over a period of 40 years) among our set of 26 tree species, with values as low as 0.001 for *Fagus grandifolia* to 6,797 for Acer *rubrum* (Table 2). The disparity between these two species is characteristic of a broader divergence in NVSGD values between broadleaf and conifer species. For conifers, values are limited to the low to intermediate classes, with NVSGD values varying between 1.4 for *Pinus resinosa* and 62.7 for *Thuja occidentalis*. One exception among conifers is *Tsuga canadensis*, which possesses a very low within‐population potential for genetic exchange with a NVSGD value of 0.6.

**TABLE 2:** Traits and values underlying genetic exchange within populations and calculation of the number of viable seeds that are genetically distinct (NVSGD) for the 26 species considered in this study

Species	Fecundity^a^ (×10^6^ viable seeds ha^−1^ 40 years^−1^)	Extent of genetic exchange			DA^a^	NVSGD (×10^6^ viable seeds ha^−1^ 40 years^−1^)	Class of genetic exchange within populations
		Mating system	Pollination vector	GM			
Conifers							
*Abies balsamea*	19.3	Outcrossing to mixed	Wind	0.75	0.7	10.2	Low
*Larix laricina*	14.3	Outcrossing to mixed	Wind	0.75	0.8	8.6	Low
*Picea glauca*	60.0	Outcrossing	Wind	1	0.8	48.0	Intermediate
*Picea mariana*	15.6	Outcrossing to mixed	Wind	0.75	0.8	9.4	Low
*Picea rubens*	53.6	Mixed	Wind	0.5	0.8	21.4	Low
*Pinus banksiana*	36.4	Outcrossing	Wind	1	0.7	25.5	Low
*Pinus resinosa*	2.4	Outcrossing to mixed	Wind	0.75	0.8	1.4	Low
*Pinus strobus*	26.2	Outcrossing	Wind	1	0.8	21.0	Intermediate
*Thuja occidentalis*	261.1	Mixed to selfing	Wind	0.3	0.8	62.7	Intermediate
*Tsuga canadensis*	1.6	Mixed	Wind	0.5	0.7	0.6	Very low
Broadleaf							
*Acer rubrum*	10,070.1	Outcrossing	Wind, insects	0.75	0.9	6,797.3	Very high
*Acer saccharinum*	273.3	Outcrossing	Wind, insects	0.75	0.8	164.0	High
*Acer saccharum*	28.4	Outcrossing	Wind, insects	0.75	0.8	17.0	Low
*Alnus incana* subsp. *rugosa*	1,227.0	Outcrossing	Wind	1	0.8	981.6	High
*Betula alleghaniensis*	95.4	Outcrossing	Wind	1	0.9	85.9	Intermediate
*Betula papyrifera*	918.6	Outcrossing	Wind	1	1	918.6	High
*Fagus grandifolia*	0.005	Outcrossing	Wind	1	0.2	0.001	Very low
*Fraxinus americana*	114.0	Outcrossing	Wind	1	0.9	102.6	High
*Fraxinus nigra*	38.9	Outcrossing	Wind	1	0.8	31.1	Intermediate
*Ostrya virginiana*	0.8	Outcrossing	Wind	1	0.8	0.6	Very low
*Populus balsamifera*	700.4	Outcrossing	Wind	1	1	700.4	High
*Populus grandidentata*	2,998.9	Outcrossing	Wind	1	1	2,998.9	Very high
*Populus tremuloides*	2,819.7	Outcrossing	Wind	1	1	2,819.7	Very high
*Prunus pensylvanica*	16.2	Mixed	Insects	0.25	0.2	0.8	Low
*Quercus rubra*	0.5	Outcrossing	Wind	1	0.2	0.1	Very low
*Tilia americana*	1.0	Outcrossing	Insects, wind	0.75	0.7	0.5	Very low

Abbreviations: DA, dispersal ability; GM, extent of genetic mixing.

^a^ Based on the method described in Boisvert‐Marsh et al. (2020). Trait values obtained from the TOPIC database (Aubin et al., 2020).

### 3.4 Component #4: Genetic exchange between populations as determined by population differentiation

**FIGURE 5:**
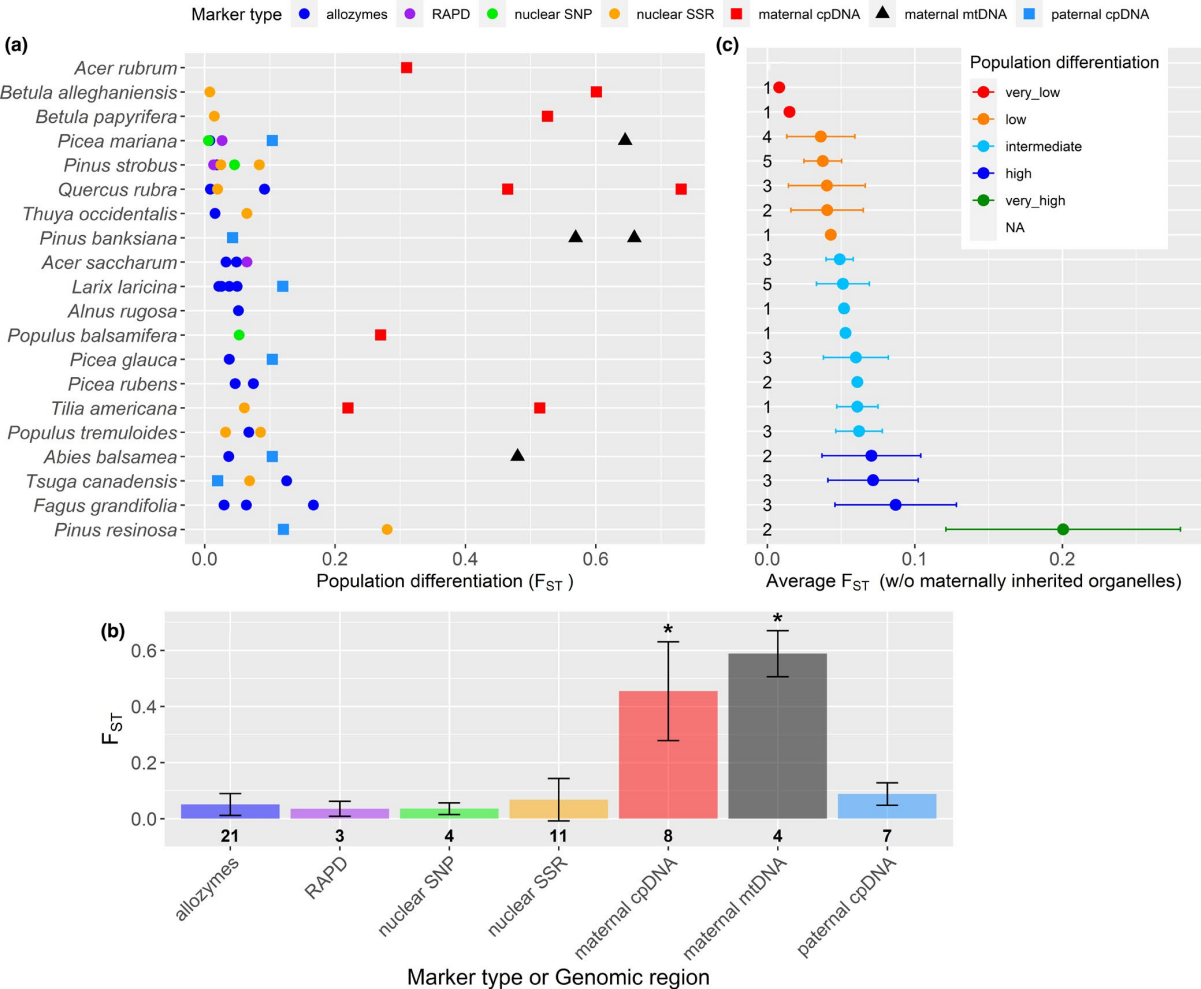
Population differentiation (*F*_ST_) for 20 of the 26 species considered in this meta‐analysis. No data were found in the literature for the remaining six species (i.e., hatched cells in the column “Population differentiation” in Figure 6). (a) *F*_ST_ values reported in the literature. (b) Average values for each method. Error bars represent the standard deviation, and numbers indicate the number of values per method. The error bar for nuclear SSR crosses zero because of the skewed distribution of the values for this marker type. (c) Average level of genetic differentiation excluding *F*_ST_ values based on maternally inherited organelles (maternal cpDNA and maternal mtDNA). For *Acer rubrum*, there were no data other than for maternally inherited cpDNA. Error bars represent the standard error. For each species (*y*‐axis), numbers on the left indicate the number of observations

There was a large intraspecific variation observed in the different *F*_ST_ estimates found in the literature (Figure 5). This variation was mainly explained by the different types of genetic markers, which were also the only significant term in our linear model (marker type SS = 1.91, species SS = 0.12, residuals SS = 0.22). Maternally inherited cpDNA and mtDNA yielded significantly higher *F*_ST_ values than allozymes (*p* < .001).

Allozyme, RAPD, SNPs related to the nuclear genome, and paternally inherited cpDNA yielded more comparable results between species. Species comparisons based on these marker types indicated that the genus *Betula* has relatively low differentiation between populations whereas *Pinus resinosa* and, to a lesser extent, *Fagus grandifolia*, *Tsuga canadensis,* and *Abies balsamea* show the highest population differentiation (Figure 5).

*F*_ST_ values measured at the regional scale tended to be lower than those measured across the entire distribution of a species when all marker types were considered (*W* = 245.5, *p* = .011), but also for the subset of data containing allozymes, RAPD, nuclear SNP, nuclear SSR, and paternal cpDNA values (*W* = 151.5, *p* = .015; details of these tests are provided in Appendix S5).

### 3.5 Component #5: Potential for genetic exchange between species through hybridization

Overall, our meta‐analysis showed the genera *Picea*, *Betula*, *Populus*, and *Quercus* particularly suited for hybridization as they can cross with many related native species (Table 3). The genus *Betula* is particularly flexible in terms of hybridization, with hybrids reported between *B*. *papyrifera* and shrub species *B. nana*, *B*. *glandulosa*, and *B*. *pumila* (Ashburner & McAllister, 2016; Burns & Honkala, 1990). Similarly, *Quercus rubra* is reported to cross with the small shrubby *Q*. *illicifolia* (Burns & Honkala, 1990). Seven species have no reported accounts of hybridization. *Larix laricina* does not form hybrids with native species but with three exotic larch species from the Eurasian continent. Because of this particularity, we classified this species as intermediate because of its potential to adapt with species moved through human‐assisted translocations.

**TABLE 3:** Reported hybrids formed between our 26 tree species and native and exotic relatives. Here, we use the term “native” for species whose original distribution includes North America; otherwise, they are referred to as “exotic”

Species	Hybridization	Mating species		References
		Native	Exotic	
Conifers				
*Abies balsamea*	Yes	*Abies lasiocarpa*		Cinget et al. (2015)
*Larix laricina*	Yes	–	*Larix decidua, L. kaempferi, L. siberica*	Burns and Honkala (1990)
*Picea glauca*	Yes	*Picea engelmannii, P. pungens, P. sitchensis*	*P. jezoensis, P. koyamai, P. omorika, P. schrenkiana*	Nkongolo et al. (2005)
*Picea mariana*	Yes	*Picea rubens*		Perron and Bousquet (1997)
*Picea rubens*	Yes	*Picea mariana*		Perron and Bousquet (1997)
*Pinus banksiana*	Yes	*Pinus contorta*		Burns and Honkala (1990)
*Pinus resinosa*	No	–	–	
*Pinus strobus*	Yes	*Pinus monticola*	*P. peuce, P. griffithii, P. parviflora, P. flexilis, P. ayacahuite*	Burns and Honkala (1990)
*Thuja occidentalis*	Yes	*Thuja plicata*		Zieliński et al. (2019)
*Tsuga canadensis*	No	–	–	
Broadleaves				
*Acer rubrum*	Yes	*Acer saccharinum*		Saeki et al. (2011)
*Acer saccharinum*	Yes	*Acer rubrum*		Saeki et al. (2011)
*Acer saccharum*	Yes	*Acer nigrum*		Dansereau and Desmarais (1947), Skepner and Krane (1998)
*Alnus incana* subsp. *rugosa*	Yes	*Alnus serrulata*		Furlow (2009)
*Betula alleghaniensis*	Yes	*Betula papyrifera, B. lenta, B. nigra, B. populifolia*	*B. pendula, B. pubescens, B. dahurica, B. platyphylla* subsp. *mandshurica*	Burns and Honkala (1990), Thomson (2013)
*Betula papyrifera*	Yes	*Betula alleghaniensis, B. lenta, B. nigra, B. neoalaskana, B. occidentalis*		Burns and Honkala (1990), Thomson (2013)
*Fagus grandifolia*	No	–	–	
*Fraxinus americana*	Yes	*Fraxinus texensis,F. pennsylvanica, F. caroliniana*	*F. mandshurica*	Burns and Honkala (1990), Wallander (2008), He et al. (2019)
*Fraxinus nigra*	No	–	–	
*Ostrya virginiana*	No	–	–	
*Populus balsamifera*	Yes	*P. deltoides, P. trichocarpa, P. angustifolia, P. fremontii*	*Populus alba, P. laurifolia, P. nigra, P. simonii, P. suaveolens, P. tremula, P. tristis*	Burns and Honkala (1990), Stettler et al. (1996), Talbot et al. (2012), Isabel et al. (2013), Suarez‐Gonzalez, Hefer, et al. (2018)
*Populus grandidentata*	Yes	*P. tremuloides*	*Populus alba*	Stettler et al. (1996)
*Populus tremuloides*	Yes	*P. grandidentata*	*Populus alba*	Stettler et al. (1996)
*Prunus pensylvanica*	No	–	–	
*Quercus rubra*	Yes	*Quercus coccinea, Q. ellipsoidalis, Q. illicifolia, Q. imbricaria, Q. marilandica, Q. palustris, Q. phellos, Q. shumardii, Q. velutina*		Burns and Honkala (1990)
*Tilia americana*	No	–	–	Burns and Honkala (1990), McCarthy and Mason‐Gamer (2016)

### 3.6 Index of adaptive capacity

**FIGURE 6:**
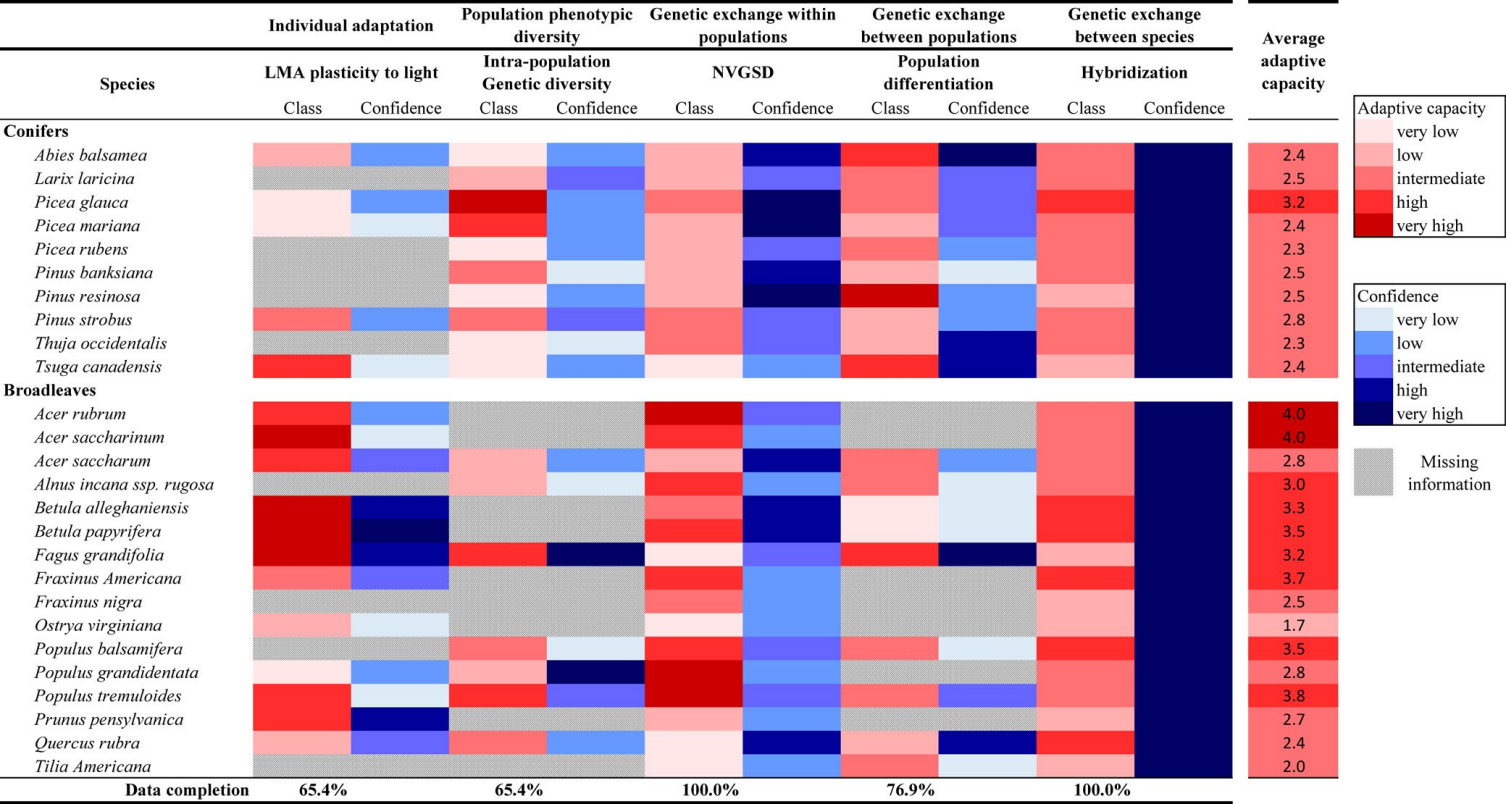
Adaptive capacity of 26 northeastern North American tree species, as characterized by the five metrics chosen to represent the five main adaptation components. Adaptive capacity for each component was ranked into five classes (e.g., *k*‐means clustering in Figures 3, 4, 5) as represented by a gradient of red tones (with dark red representing high adaptive capacity). Confidence levels associated with available data are represented by blue tones (with dark blue representing high confidence level). Confidence level for LMA plasticity to light, genetic diversity, and population differentiation was based on the standard error of numerical values and clustered into four equally sized classes. A fifth class was attributed to singletons and depicts the lowest level of confidence. For NVSGD, the confidence level represents average of confidence levels provided by Boisvert‐Marsh et al. (2020) based on the availability of trait data for reproductive capacity and dispersal ability mechanisms. For hybridization, confidence level was considered high for each observation supported by a reference in Table 3

Our set of indices outlines important variation in adaptive capacity among our 26 species (Figure 6). Important variation is also observed across the five components of adaptation such that no one species ranks consistently across all components. On average, *Picea glauca* and *Acer rubrum and Acer saccharinum* are considered the most adaptive conifer and broadleaf species in our study. Other broadleaf species such as *Populus tremuloides*, *Betula* *papyrifera*, and *Fraxinus americana* also have a high overall adaptive capacity. Conversely, *Picea rubens* and *Thuja occidentalis,* and *Ostrya virginiana* are classified as the least adaptive conifers and broadleaf species, respectively.

Confidence level in this classification varies greatly between components of adaptive capacity, highlighting differences in the quality of data available. Hybridization capacity and NVSGD index (our proxy for the genetic exchange within population) are well documented and exhibited the highest confidence levels. Conversely, LMA plasticity to light as well as within‐ and between‐population genetic diversity (*H*_s_ and *F*_ST_) metrics have lower levels of confidence for most of the documented species. Confidence levels also vary between species. Data confidence for LMA plasticity to light is generally higher in broadleaf species than in conifers. Conversely, the confidence level for NVSGD and *F*_ST_ tends to be higher for conifers than for broadleaf species.

Important variation in data availability is found between components and across species. At the species level, data completeness for our 26 tree species averages 77%; this proportion is higher for conifers than for broadleaf species (86% vs. 71%) but varies considerably across species (Figure 6). Data completeness also varies greatly between components of adaptive capacity, with the NVSGD index, used to characterize genetic exchange within population, and hybridization capacity being documented for 100% of our study species. Conversely, LMA plasticity to light and Intra‐population genetic diversity were the least documented components of adaptive capacity, with only 65.4% of species being documented. Data completeness for the indices used to characterize population phenotypic diversity and genetic exchange between populations is also higher for conifer species (100% species documented) than broadleaf species (44% and 56% species documented, respectively). For LMA plasticity to light, used here as a metric of individual adaptation, more broadleaf species than conifers are documented (75% vs. 50% species documented, respectively, Appendix S2: Table A2.1).

## 4 DISCUSSION

Adaptive capacity is an important but “elusive” (Scotti, 2010) determinant of tree capacity to persist in situ (Aitken et al., 2008; Aubin et al., 2016) as it manifests in multiple processes (biological, physiological, ecological, and evolutionary) that act across a range of temporal and spatial scales. Adaptive capacity remains the least understood and least documented determinant of vulnerability to climate change, notably because the data needed to quantify it are complex and heterogeneous in nature (de los Ríos et al., 2018; Thurman et al., 2020). In this paper, we developed a transdisciplinary framework to characterize tree adaptive capacity into five key components. This framework adopts elements from both the comprehensive approach to adaptive capacity—that is, focusing on selective forces acting on a single species or a few related ones—and the comparative, multispecies approach—that is, integrating refined metrics of phenotypic and genotypic features of species and standardizing them for cross‐comparison purposes.

Using this framework, we developed a set of five indices, one for each component of adaptive capacity, and performed a meta‐analysis to select indices to document the adaptive capacity of 26 abundant tree species in northeastern North America. The use of a set of indices instead than a single catch‐all index provides a comprehensive and flexible format to capture tree adaptive capacity. Each index depicts the various pathways across space and time by which species’ adaptation to climate change could occur. The advantage of this approach is that the user can select one or more indices according to their specific context. Using this flexible framework, new information can be added as data become available and new species can be documented, such as when applying this approach to other regions of the world, or when documenting other tree species of interest.

From a forest management perspective, our indices provide insight into species’ adaptive potential in the short (Components 1 and 2) and longer terms (Components 3–5), both of which are important for planning future reforestation programs and climate change adaptation actions. For example, species such as *Betula alleghaniensis* and *Fagus grandifolia* showed a high level of leaf plasticity to light, highlighting their capacity to adapt to different light environments. Promoting a diversity of phenotypically plastic species in forests could provide a buffer against short‐term changes in climate‐related environmental conditions, such as changes in forest structure and canopy gaps caused by tree mortality (Goulet & Bellefleur, 1986) or variations in water availability (Ramírez‐Valiente et al., 2010).

Diversity can also be promoted by favoring and maintaining high levels of intrapopulation genetic variation, which in turn could affect how species respond to adverse conditions. For example, species distribution models for *Picea glauca* predict a strong reduction in its climate niche in southern Canada by the end of the century (McKenney et al., 2011; Périé et al., 2014). However, models based solely on climate do not consider the considerable levels of genetic diversity currently held within natural populations. Hence, *Picea glauca* may possess higher capacity to adapt and persist depending on the area under consideration. Recently, Depardieu et al. (2020) found substantial variation in wood traits and drought resilience among *Picea glauca* provenances and families that could be harnessed by tree breeders to select for a range of potential climate change outcomes. Conversely, the consequences of low levels of genetic diversity, as in important commercial species such as *Pinus resinosa,* should be considered in management plans.

Species with a high capacity to colonize newly available areas and fast generation times, such as *Acer rubrum*, possess a higher capacity to exchange genetic material within populations and should therefore cope well under climate change (Boisvert‐Marsh et al., 2020). Evidence of range expansion into the southern boreal forest has already been observed for this species (Boisvert‐Marsh & Blois, 2021; Brice et al., 2019), trends which are expected to continue over the next century (Bouchard et al., 2019; Boulanger et al., 2017). While typically a nontarget species in silviculture, new approaches may need to incorporate such climate‐adaptive species into forest management planning of stands susceptible to rapid shifts in species composition.

High potential of genetic exchange between populations via genetic differentiation may offer opportunities for human‐mediated translocation of individuals between populations or for breeding better adapted individuals (Girardin et al., 2021). For instance, high differentiation between populations of *Fagus grandifolia* may hold promise in the search for individuals resistant to beech bark disease, an insect–fungus complex that is decimating beech populations and altering ecological successional pathways in temperate forests (Roy & Nolet, 2018). Depending on its direction, natural gene flow between genetically distinct tree populations may also be beneficial for climate change adaptation (Godbout et al., 2020).

The capacity to exchange genetic material between species could provide an overlooked opportunity in the face of climate change. Hybridization has been shown to provide an adaptive advantage in the past (Kremer & Hipp, 2020) and could play an important role in the persistence of genera with a strong ability to form hybrids (Menon et al., 2020). For certain species, hybridization ability has already been used to produce more productive hybrids and clones (e.g., *Populus* spp. Zhang et al., 2003). In a context of climate and global changes, hybridization could be further used to produce trees that are resistant to pests (Westbrook et al., 2020) or drought (Zeng et al., 2014).

This work may also be useful for modelers, providing a framework and data to parameterize species distribution models (SDMs) when exploring potential impacts of climate change. Studies on the genetic variation underlying climate tolerance are useful to improve model fits of current and future suitable distribution (reviewed in Peterson et al., 2019). For example, models by Benito Garzón et al. (2019) delineated regions of high and low survival probability in areas expected to become unsuitable within current species range by integrating tree phenotypic plasticity and local adaptation into SDMs. Our indices could extend these assessments across multiple species by comparing their relative adaptive capacity in areas projected to become newly suitable or unsuitable (Willis et al., 2015). Individual components of our framework could be useful in specific contexts. For instance, the NVGSD index used to characterize genetic exchange within populations could provide standardized data to models that use generation time, propagule pressure, and pollen/seed dispersal ability to estimate rates of genetic mixing and selection (Bush et al., 2016; Huey et al., 2012). Metrics of population differentiation are also useful to better understand how gene flow, dispersal barriers, and selective pressures interact to influence the strength of evolution and adaptation (Alberto et al., 2013; Scoble & Lowe, 2010). Work on understanding inheritance and selection from tree breeding efforts also hold promise for use in modeling, but more work is needed in natural populations before they can be fully incorporated (Alberto et al., 2013).

## 5 LIMITATIONS AND THE WAY FORWARD

Our classification synthesizes current knowledge on interspecific differences in species’ adaptive capacity, a classification that is consistent with the literature. For instance, our index on LMA plasticity to light (Component 1) is consistent with the classification of Goulet and Bellefleur (1986), who observed greater plasticity in the shade‐tolerant *Fagus grandifolia* and moderately tolerant *Betula alleghaniensis* compared with less shade‐tolerant tree species. Additionally, a low level of genetic diversity has often been reported for *Pinus resinosa* (Hamrick et al., 1992; Mosseler et al., 1992). However, data availability and comparability pose considerable challenges to the comparative multispecies approach to adaptive capacity.

### 5.1 Data availability

Among the greatest challenges in the development of these indices was data availability, particularly within the indices of phenotypic plasticity, genetic diversity, and population differentiation (components 1, 2, and 4, respectively). Even for LMA, which is one of the most sampled and readily accessible plant traits studied (Kattge et al., 2020), LMA data were lacking for 9 of our 26 species. Across indices, we found that commercial tree species had the most data, both in availability and in quantity, while a large number of common tree species were left undocumented (e.g., *Fraxinus nigra* and *Tilia americana;* gray in Figure 6). Documenting adaptive capacity across a broad range of species (~320 species in North American temperate forests alone; Huntley, 1993) is necessary to accurately anticipate forest response to climate change. Therefore, the paucity of data should be a call to the scientific community to broaden the scope of studied species beyond commercial ones.

### 5.2 Data comparability

Data comparability issues caused by variation in methodology also affected index development. Although routinely used in comparative ecology (e.g., functional ecology Díaz et al., 2016; Lavorel, 2013), substantial variation can be introduced into measurements of ecophysiological traits based on the choice of standards and/or units used, the life stage of the sampled individual (seedling or mature tree), and the measurement location (e.g., stems or twigs; Aubin et al., 2016). For example, LMA measurements can be influenced by whether or not the leaf petiole is included, a choice that largely depends on the initial study objectives (Pérez‐Harguindeguy et al., 2013). In the studies we found, 15% included the petiole in LMA measurements, 50% excluded it and 35% did not specify. Similarly, most LMA measurements used in our meta‐analysis (42 out of 73) were conducted on seedlings, with the rest being evenly divided between saplings and mature trees.

Comparisons across species were also complicated by the different genetic markers used across the various studies and their specific particularities (Ai et al., 2014; Hall & Beissinger, 2014). For instance, SSR loci commonly have multiple alleles (e.g., 16–18 different alleles in Godbout et al., 2010). This high allelic richness statistically inflates genetic diversity measures compared with other marker types for which only a few alleles are present. For example, SNPs in diploid organisms are commonly biallelic markers and thus, because of how expected heterozygosity (*H*_e_) is calculated, cannot present values higher than 0.5 (see Jost, 2006 for more details). This statistical property of heterozygosity might also explain why genetic diversity estimates based on haplotypes yielded higher values compared with allozymes and other methods (see Appendix S2 for details of haplotype diversity calculation). Haplotype sequences contain many variable sites (SNPs, indels, or SSR) for which different combinations are possible, thereby yielding multiple alleles (Pavy et al., 2012). As well, certain markers may amplify natural variation, even for the set of markers that we used in this study. RAPD genetic markers are dominant markers—that is, only one band profile is expressed—even for heterozygotes, possibly amplifying estimations of *H*_s_ artificially. A correction can be applied based on the proportion of recessive individuals in a population (Lynch & Milligan, 1994), but this correction has not been done systematically. Within our set of species, RAPD values for *Acer saccharum*, *Pinus strobus,* and *Picea mariana* were in fact corrected, but those for *Populus grandidentata* and *P*. *tremuloides* were not. This situation might have amplified the observed variation for these *Populus* species.

For population differentiation (*F*_ST_, component 4), the large level of variation observed between marker types was related to the parental inheritance of the genetic material. Typically, mtDNA is maternally inherited in both conifers and angiosperms while cpDNA is paternally inherited in conifers and maternally inherited in angiosperms (but many exceptions to this general pattern exist, see Hagemann, 2004). For our subset of species, maternally inherited organelles showed higher *F*_ST_ than paternally inherited ones (Figure 5). Because seeds are generally dispersed over smaller distances than pollen, maternally inherited markers preserve the imprints of large historical events (such as glaciation) on population differentiation better than paternally inherited markers.

### 5.3 Potential avenues and solutions

Together, the aforementioned limitations call for the collection of additional data from a wide range of environmental conditions and locations across a species' distribution and with cross‐comparability between species in mind (Kattge et al., 2020). Advances in sequencing technologies (e.g., RADseq; Andrews et al., 2016) may provide effective solutions to rapidly survey the genetic diversity of multiple species at once. Additional solutions may reside in adopting some methods from the comprehensive approach to measure adaptive capacity. In the case of phenotypic plasticity, for instance, reaction norms—curves that describe the range of trait values expressed by a single genotype across an environmental gradient (e.g., Rehfeldt et al., 2018)—may provide more precise estimates of phenotypic plasticity (Arnold et al., 2019). This approach, however, requires phenotypic and fitness measurements from multiple genotypes grown in different environments that have yet to be conducted for many tree species.

### 5.4 Some of the challenges ahead

The work presented here represents a first step toward characterizing tree adaptive capacity, and important challenges remain before we can translate these indicators into robust metrics of species’ ability to persist in situ.

- *To what extent the observed level of phenotypic plasticity is adaptive*? Plant fitness should be assessed in conjunction with phenotypic plasticity to identify and quantify adaptive plasticity. Similarly, h*ow many traits are needed to adequately capture adaptive capacity*? The ranking of species plasticity may differ depending on the trait considered because some traits are more variable than others and whether or not the traits considered covary (Auger & Shipley, 2013). However, such multitrait evaluations of species’ plasticity are currently not feasible because data gaps considerably reduce the number of species that can be compared at once.
- *How much genetic variation is required for adaptation*? Obviously, there appears to be a trade‐off between beneficial and harmful aspects of genetic diversity, as exemplified by the balance between adaptive introgression and outbreeding depression (Suarez‐Gonzalez, Lexer, et al., 2018). These counteracting principles should be better understood before we can determine how much gene flow is required to resolve important issues, such as ensuring population survival or the number of seed sources that should be used in a locality.
- *What is the role of epigenetic and other mechanisms in adaptive capacity?* For many conifer species, epigenetic mechanisms are thought to play a role in the rapid adaptation of seedlings in novel environments (Yakovlev et al., 2012). In the context of climate change, epigenetic modifications found in natural tree populations could rapidly enhance their capacity to persist in place (Grant‐Downton & Dickinson, 2006; Thiebaut et al., 2019) and are hypothesized to have enabled clonal species to survive past environmental changes (Dodd & Douhovnikoff, 2016). However, we still do not know to what extent epigenetics are prevalent in tree populations, since most studies have focused on only a few genera (*Picea*, *Pinus* and *Populus*; Bräutigam et al., 2013; Guarino et al., 2015; Le Gac et al., 2018; Yakovlev et al., 2012). Similarly, we need more studies on other possibly important factors, such as polyploidy (e.g., Mock et al., 2012), mating system plasticity (Peterson & Kay, 2015), and clonal reproduction (Dodd & Douhovnikoff, 2016) to integrate them in tree species adaptive capacity assessments.

## 6 CONCLUSION

Variability is a key concept of adaptive capacity, from the variability in the phenotype that can be expressed by a single individual to the genetic variation between individuals and populations. Yet, biologists have long sought to categorize natural variability into well‐defined groups such as species, ecotypes, and populations. As a result, our knowledge of tree species is based on averages, with variability being relegated to the category of “noise” in the data that needs to be controlled for (Bradshaw, 2006; Shipley et al., 2016). In the context of global changes, this perspective about biological diversity may be obsolete. As we have demonstrated, embracing variability instead of ignoring it (Violle et al., 2012) is an important step toward improving our understanding of tree responses to these changes.

The breadth of data required to document the five components exemplifies that adaptive capacity is indeed a multidisciplinary concept (e.g., ecophysiology, ecology, population genetics, and conservation). This means that the study of adaptive capacity inevitably requires collaboration and data sharing between experts from different disciplines. The shift toward “open science” has dramatically increased data sharing and interdisciplinary collaborations in ecology, generating a renewal in the scope and scale of studies (Aubin et al., 2020). Continuing these efforts and extending collaboration across seemingly disparate fields may be the way to fully grasp tree adaptive capacity, further our capacity to anticipate tree species persistence in situ and assist the development of climate adaptation strategies.

## CONFLICT OF INTEREST

The authors declare no conflict of interest.

## AUTHOR CONTRIBUTIONS

**Samuel Royer‐Tardif:** Conceptualization (equal); Data curation (equal); Formal analysis (lead); Investigation (lead); Methodology (lead); Project administration (lead); Validation (equal); Visualization (lead); Writing‐original draft (lead); Writing‐review & editing (lead). **Laura Boisvert‐Marsh:** Conceptualization (equal); Data curation (equal); Formal analysis (supporting); Investigation (supporting); Methodology (supporting); Validation (equal); Visualization (supporting); Writing‐original draft (supporting); Writing‐review & editing (supporting). **Julie Godbout:** Formal analysis (supporting); Investigation (supporting); Methodology (supporting); Validation (equal); Visualization (supporting); Writing‐original draft (supporting); Writing‐review & editing (supporting). **Nathalie Isabel:** Methodology (supporting); Resources (equal); Validation (equal); Writing‐original draft (supporting); Writing‐review & editing (supporting). **Isabelle Aubin:** Conceptualization (equal); Formal analysis (supporting); Funding acquisition (lead); Investigation (supporting); Methodology (supporting); Project administration (supporting); Resources (equal); Supervision (lead); Validation (equal); Visualization (supporting); Writing‐original draft (supporting); Writing‐review & editing (supporting).

## Supporting information

Figure S1

Appendix S1‐S5

## Data Availability

The list of studies used to build Table A2.1 is provided in Table S1. LMA data used in the calculation of plasticity indices and their source are provided in Table S2. All genetic parameters used in this study are presented in Table S3 (*H*_e_, *F*_ST_, *D*, *F*_IS_). Tables S1, S2, and S3, associated metadata, and the complete list of references are deposited in the Dryad Digital repository: https://doi.org/10.5061/dryad.76hdr7sx5. Data associated with NVSGD computation have been deposited in the TOPIC database (Aubin et al., 2020) and are freely available upon request to the database.
