# Supplementary figures and images for: Finding common ground: Toward comparable indicators of adaptive capacity of tree species to a changing climate
Source: Ecol Evol. 2021 Sep 2;11(19):13081–100. doi: 10.1002/ece3.8024 (PMC8495821; doi:10.1002/ece3.8024)

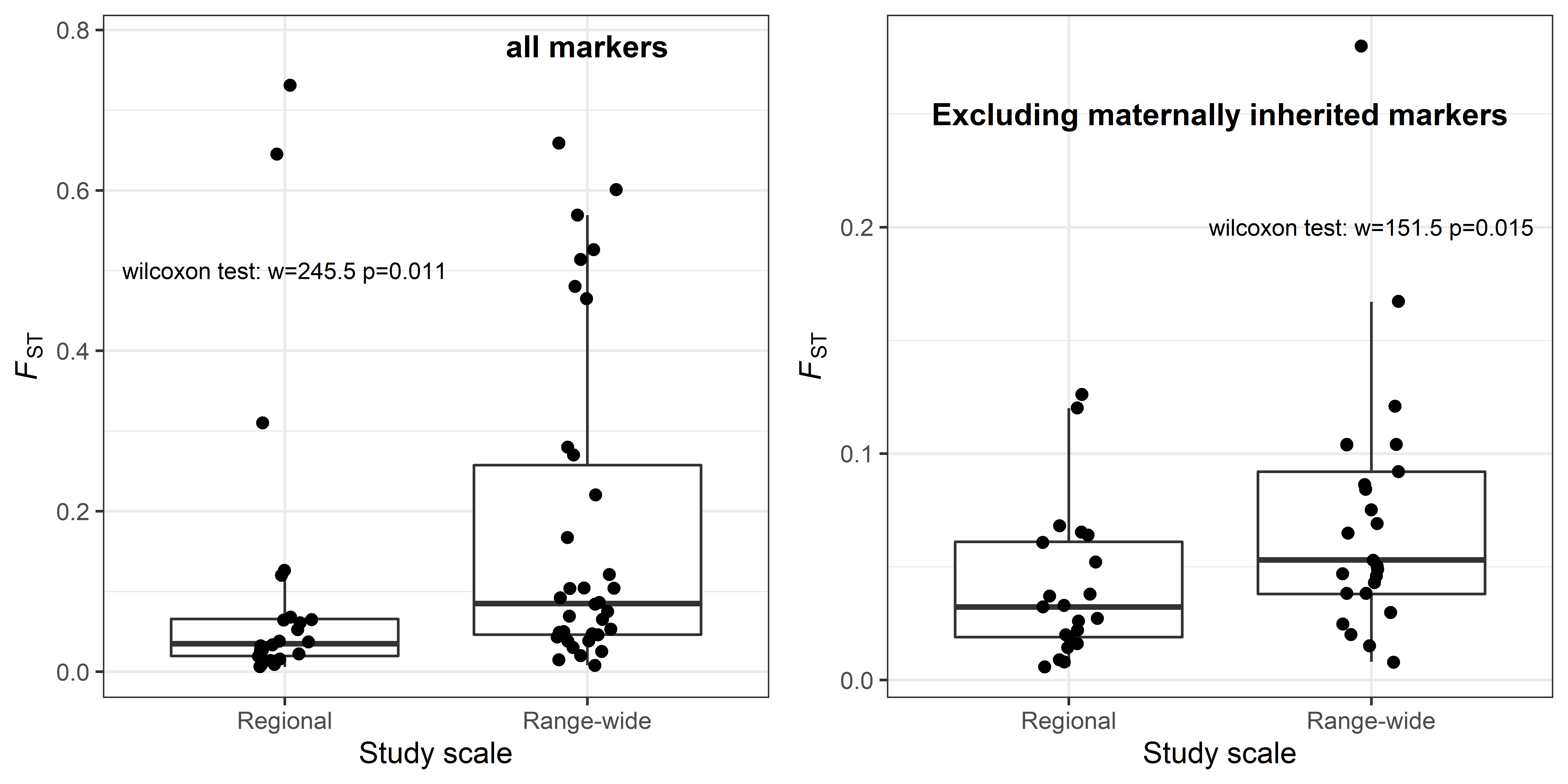

Supplement: Supplementary file 1 — Figure S1 [file ECE3-11-13081-s002.tiff]
