# Supplementary material for: Finding common ground: Toward comparable indicators of adaptive capacity of tree species to a changing climate
Source: Ecol Evol. 2021 Sep 2;11(19):13081–100. doi: 10.1002/ece3.8024 (PMC8495821; doi:10.1002/ece3.8024)
Supplement: Supplementary file 2 — Appendix S1‐S5 [file ECE3-11-13081-s001.docx]

# APPENDICES

# Appendix S1: Rationale behind each component of adaptive capacity

## Component #1: Individual adaptation through phenotypic plasticity

At the scale of an individual, trees can cope with novel climatic conditions by modulating their phenotype, i.e., their morphology and physiology (Chmura et al. 2011). This ability is called phenotypic plasticity, and is defined as the capacity of a single genotype (i.e., a unique set of genes) to express different phenotypes under different environmental conditions (Nicotra et al. 2010). Phenotypic plasticity may be especially important for the persistence of sessile, long-lived organisms, such as trees, under climate change (Alfaro et al. 2014). For example, plasticity in the timing of bud burst is thought to play an important role in plant phenological responses to earlier warming in the spring (Franks et al. 2014). Conversely, some authors have argued that plasticity could delay species’ evolutionary adaptation as it allows maladapted genotypes to persist despite a changing environment (Ghalambor et al. 2007, Aitken et al. 2008). However, empirical evidence does support the idea that plastic and evolutionary responses are not mutually exclusive, and can occur simultaneously (Franks et al. 2014). Moreover, plasticity in itself is an adaptive attribute with a genetic component that can evolve in variable environments (Bradshaw 1965, Nicotra et al. 2010).

### Comparing levels of phenotypic plasticity

Which tree species are the most plastic? This simple question has been explored extensively in comparative studies of temperate trees (e.g., Goulet and Bellefleur 1986, Lei and Lechowicz 1998, Sanford et al. 2003, Paquette et al. 2007, 2012). However, these studies focused on only a few species at a time such that a complete picture of comparative plasticity among tree species is still lacking. In addition, it is difficult to compare plasticity values taken from different studies and species due to multiple confounding factors, including the different organisation scales at which phenotypic plasticity can be measured, the diversity of indices used, and gaps in data availability. In recent decades, several important reviews have highlighted these issues and suggested ways to overcome them (Valladares et al. 2007, Nicotra et al. 2010, Arnold et al. 2019).

### Issues when comparing phenotypic plasticity

Comparisons of levels of phenotypic plasticity between species are complicated by many factors. First, different studies measure phenotypic plasticity at different scales of organization (Bradshaw 1965, Ghalambor et al. 2007, Lande 2009, Gianoli and Valladares 2012). In studies that explore its genetic controls, phenotypic plasticity tends to closely follow its formal definition (Nicotra et al. 2010), and is limited strictly to the phenotypes expressed by a single genotype (e.g., Bradshaw 1965, Ghalambor et al. 2007, Lande 2009). This implies that these studies necessarily grow plants with the same genotype (i.e., clones) in different environments/locations. By comparison, in ecological studies, plasticity is sometimes extended to include the variation expressed by genetically related individuals, such as families, populations or even entire species (e.g., the role of plasticity in exotic and invasive plant success: Palacio‐López and Gianoli 2011, Paquette et al. 2012). This broader definition has its advantages: it is applicable to species for which clonal propagation is limited, enables *in situ* observational studies and increases potential sample size. Therefore, it increases the range of environmental conditions covered and the statistical power of studies (Gianoli and Valladares 2012). However, expanding the concept of phenotypic plasticity creates comparability issues across studies focusing on different organisation levels (e.g., within-individual variation vs within-species) and confounds individual adaptation and population phenotypic diversity (see Figure 2). For many species, however, data availability issues limit quantifying the variation within individuals, such that only variation within populations or species are available to assess phenotypic plasticity.

Second, the various indices that capture phenotypic plasticity are often not comparable amongst each other without having access to the original data. Valladares et al. (2006) reported 17 of these indices (e.g., coefficient of variation, slope of norm of reaction) and found that the majority (14 of 17) could not be compared across species and studies. The breadth of environmental conditions surveyed may also influence comparisons between studies and species. To be ecologically meaningful, evaluations of phenotypic plasticity should explore the complete range of environmental conditions experienced by a species (Valladares et al. 2007), which is likely to differ from one species to another. For example, tree species have different ranges of light intensity under which they can grow. A flexible and comparable plasticity index should therefore standardize trait variation based on the change in trait values across a range of environmental conditions experienced by the same individual. However, phenotypic variation may not be related linearly to changes in environmental values (e.g., change in light availability; Valladares et al. 2006), further complicating species comparisons across different studies.

Data gaps also complicate comparisons of plasticity among tree species. This issue is well known to database curators: plasticity is measured on a large suite of plant traits that are not assessed consistently across studies and species, such that the number of species with good trait coverage decreases quickly with the number of traits considered (Kattge et al. 2020). Moreover, not all trait variation is necessarily adaptive in a changing environment, i.e., leads to increased individual fitness (Ghalambor et al. 2007). The comparison of adaptive plasticity should therefore focus on frequently measured traits for which the intra-individual variation depicts adaptive responses in a changing climate.

A list of such key traits was provided by Nicotra et al. (2010) and included traits related to leaf morphology, reproduction rate, phenology and plant size as priority traits to investigate. For the purpose of this study, we excluded tree height at maturity (or height at reproduction) because determining their plasticity is difficult because of the longevity of most species.

## Component #2: Population phenotypic diversity as influenced by genetic diversity

Over time, climate change is expected to exceed phenotypic plasticity (Gienapp et al. 2008). At the scale of a population (i.e., a group of interbreeding individuals of a species within a geographic location), the number of different phenotypes may increase the diversity of responses to climate change and thus the chances that some individuals will persist and reproduce. Therefore, populations with larger phenotypic variation stand a better chance of containing individuals that can tolerate novel conditions (Jump et al. 2009).

If phenotypic plasticity is responsible for phenotypic variation at the individual level, genetic diversity contributes to phenotypic variation at the population/species level (at least for heritable traits). It is therefore expected that higher levels of genetic diversity in plant populations should favour higher diversity of responses to environmental stress and disturbances, allowing populations to persist locally under climate change (Jump et al. 2009). Moreover, genetic diversity is the primary material for adaptive evolution (Kokko et al. 2017), and there is a general consensus that populations with higher genetic variability will also have a greater chance to adapt to novel climatic conditions (Aitken et al. 2008, Alberto et al. 2013, Alfaro et al. 2014, Bussotti et al. 2015). In general, tree species have retained high levels of genetic diversity within their populations due to a high rate of outcrossing favoured by wind-dispersed pollen and their large population size (Hamrick 2004).

Although it is central to the concept of adaptive capacity, the level of genetic diversity has seldom been compared across many tree species at once (e.g., Hamrick et al. 1992, Loveless 1992, Kremer et al. 2005, Alberto et al. 2013, Lowe et al. 2018). It is also challenging to compare measures of genetic diversity that are derived from different studies using different types of markers or different indices of genetic diversity. Here, we provide an overview of these aspects and how we dealt with them in our study.

### Adaptive vs neutral genetic diversity

Genetic diversity is divided between adaptive and neutral components, a distinction that depends on the pressure exerted by natural selection (Holderegger et al. 2006). Adaptive genetic diversity determines the fitness of individuals in their environment and is thus under selection (Hoffmann et al. 2017). Conversely, neutral genetic diversity does not seem to influence individual fitness and adaptive capacity (Holderegger et al., 2006); this means that, in the parental generations, genetic diversity was solely influenced by neutral evolutionary forces, i.e., mutations, genetic recombination, gene flow, and genetic drift.

Currently, most available metrics of genetic variation are interpreted under the premise that they represent neutral genetic diversity, unless evidence says otherwise (Isabel et al. 1995, 1999). This is related to the fact evaluating “adaptive genetic diversity” *per se* is complex, as it requires a good knowledge of the relationship between traits, genes and individual fitness (Vitti et al. 2013, Kokko et al. 2017). Unfortunately, the genetic basis for adaptive traits is not fully understood (e.g., Mahony et al. 2020, Menon et al. 2020). Natural selection may itself be variable through time and space, such that different sets of traits may be favoured through time or at different locations, thus involving different sets of genes (Kremer et al. 2014). Furthermore, the adaptive quality of some genetic variation may be cryptic in most situations, i.e., it may not contribute to phenotypic variation until the environment has been altered (Schlichting 2008, Paaby and Rockman 2014). In the context of unprecedented global changes, it is not possible to predict every potential source of selective pressure and, therefore, it is equally difficult to distinguish between future adaptive and neutral genetic variation. Therefore, a more pragmatic option is to assume that the value of genetic diversity will be proportional to its quantity (Ledig 1986).

### Types of molecular markers to evaluate neutral genetic diversity.

Since the first studies of genetic variation in tree species, various types of molecular markers and technologies have been developed (reviewed in Agarwal et al. 2008, and Mondini et al. 2009). From the use of allozymes to the study of the whole genomes, tree genetic diversity has been intensely scrutinized in order to support conservation and management decisions (Allendorf 2017).

Our meta-analysis approach, which compares the adaptive capacity of multiple species at once, implies that we need to compare genetic diversity measures obtained from different methods. While older techniques rely on the indirect assessment of the genetic variation (e.g., Restriction Fragment Length Polymorphism (RFLP), Amplified Fragment Length Polymorphism (AFLP) and Randomly Amplified Polymorphic DNA (RAPD)), more recent sequencing technologies allow for direct measurements of variation in DNA (e.g., Single Sequence Repeats (SSR, also called microsatellites), Single Nucleotide Polymorphism (SNP) or sequences). A large portion of our knowledge of tree species genetic diversity comes from older, indirect genotyping methods, such as allozymes, RFLP, AFLP and RAPD. The development of Next Generation Sequencing (NGS) technologies has further reduced the cost of sequencing-based methods such that SNPs can now be identified directly by comparing the genetic sequences obtained through NGS between different individuals of the same species. Nevertheless, the biggest difference between then and now is the quantity of information that can be generated, i.e., the number of genomic regions that can be sampled at the same time and their features (coding vs non-coding, intron, promoter, etc.).

Three different DNA genomes - nuclear, mitochondrial, and chloroplast - coexist in plants. The genetic diversity of these last two organelles present patterns that differ from those observed in nuclear DNA (haploid vs diploid) because of their specific mode of inheritance and dispersion. Indeed, mtDNA and cpDNA are generally inherited from a single parent and, depending if they are maternally or paternally inherited, they will be dispersed through seeds for the former or through pollen (then seeds after the pollination) for the latter (see Petit et al. 2005 for discussion). Because the haploid DNA molecule does not recombine (unlike the nuclear genome), it can be considered as equivalent to a single locus (a chlorotype and mitotype for cpDNA and mtDNA respectively). Improved understanding of these inherent characteristics has been closely linked to the development of the field of phylogeography (Allendorf 2017). The genetic diversity present in these organelles can be evaluated using many of the same techniques employed to evaluate the nuclear genome.

### Measuring genetic diversity

Different indices can be used to estimate population genetic diversity. Allelic richness (*A*) represents the number of different alleles (i.e., different version of the same gene) in a population. Heterozygosity (*H*), another measure of genetic diversity, corresponds to the proportion of heterozygotes in a population, i.e., individuals that bear non-identical alleles at one or more loci (e.g., genes). Heterozygosity can be directly measured in a population sample; in this case, it is called “observed heterozygosity” and is denoted as *H*_o_. Conversely, genetic diversity can also be expressed as a probability, i.e., the heterozygosity that would be expected under the Hardy-Weinberg equilibrium (i.e., in the absence of evolutionary influences). This expected heterozygosity (*H*_e_) is the allelic richness weighted by the frequency of each allele in a population, according to the formula *1-∑p_i_^2^*, where *p_i_* is the proportional frequency of the *i*th allele. In terms familiar to community ecologists, *H*_e_ corresponds to the Gini-Simpson entropy index of allelic proportional frequencies (Jost 2008). Contrary to *H*_o_, *H*_e_ is purely statistical and is not influenced by non-random mating or other factors limiting or favouring heterozygotes (Freeman and Herron 2014). Therefore, it represents a good estimator of genetic diversity that is comparable across species experiencing different conditions that influence demography.

## Component #3: Genetic exchange within populations as a function of life history traits

Eventually, the continued effects of climate change are expected to surpass the extent of phenotypic plasticity/diversity of tree populations (St Clair and Howe 2007, Savolainen et al. 2007). In this context, tree species will need to evolve if they are to persist within their current range (Aitken et al. 2008). Evolution can occur rapidly under high selective pressure in species with short generation times, such as annual plants (Kremer et al. 2014, Gallien et al. 2016, Lustenhouwer et al. 2018). , On the other hand, trees are expected to evolve over longer time scales because of their long generation times (several decades) (Alfaro et al. 2014). In addition, species-specific characteristics may cause differences in adaptive rates. For instance, species that produce more offspring over a shorter time period have more opportunities to exchange genes and a higher chance to yield genotypes adapted to future climates (Aubin et al. 2016). Indeed, through the process of genetic recombination, sexual reproduction might yield novel allelic combinations that could increase a species adaptive capacity (Rice 2002, Stapley et al. 2017). Moreover, increasing genetic mixing within populations reduces the strength of inbreeding depression (which can be strong in isolated populations) and its associated negative consequences for population viability (Charlesworth and Charlesworth 1987).

We identified three characteristics related to tree life history traits that favour genetic exchanges within populations. First, we quantified tree fecundity, which is influenced by traits such as the age at which trees first start producing seeds (i.e., age of sexual maturity) and the number of viable seeds produced once mature (Boisvert-Marsh et al. 2020). Second, we related reproductive output to the potential for genetic mixing, i.e., that resultant progeny is produced from unrelated parents. This rate of mixing is influenced by the prevalence of self-fertilization (Peterson and Kay 2015) and by pollen dispersal vector (e.g., wind disseminates pollen farther than insects). Third, we also included seed dispersal distance and dispersal vector because it can also contribute to genetic exchange by promoting gene flow within populations (Savolainen et al. 2007). Greater seed dispersal ability also increases the range of environmental conditions available for seeds and the probability of landing in a suitable habitat (Ozinga et al. 2004).

## Component #4: Genetic exchange between populations as determined by population differentiation

Gene flow could contribute to species adaptive capacity when better-suited alleles are transferred between populations. The extent of gene flow depends on population connectivity and the presence of exogenous and endogenous pre- and post- reproductive barriers (e.g., topographic barriers like mountains or rivers and biological barriers such as flowering phenology). Adaptive gene flow may occur naturally in tree populations when the direction of gene flow coincides with environmental change (Godbout et al. 2020).

The presence of distinct sources of genetic diversity throughout a species distribution may offer unique possibilities to develop translocation or breeding programs in order to improve a species’ adaptive capacity (Aitken and Bemmels 2016, MacLachlan et al. 2017). This is particularly true at the leading edge of a species’ range since trailing edge populations could be a source of alleles that are better adapted to warmer growing conditions (Fady et al. 2016).

## Component #5: Potential for genetic exchange between species through hybridization

Genetic exchange can occur between interfertile species through hybridization and may lead to introgression, i.e., the incorporation of alleles from one species’ gene pool into another. Such novel allelic assemblages can generate unique phenotypes beyond the phenotypic variation currently exhibited by parental species due to complementary allelic effects (Lexer et al. 2004) and possibly accelerate evolutionary processes (Hoffmann and Sgrò 2011). Although some of these novel phenotypes may be maladapted, hybridization also increases the odds of yielding tolerant genotypes, thereby increasing adaptive capacity (Arnold and Kunte 2017). This may be a particularly important mechanism under climate change because populations will be exposed to conditions for which preceding generations were not selected.

Natural hybridization is frequent, although unevenly distributed, among plant genera (Ellstrand et al. 1996) and occurs in regions where the distributions of two or more closely related species overlap (i.e., sympatric zones). Natural hybridization zones have been well documented for several tree genera, like *Pinus* (Rweyongeza et al. 2007, Menon et al. 2020), *Picea* (Perron and Bousquet 1997, Hamilton et al. 2014), *Quercus* (Lind-Riehl and Gailing 2017, Kremer and Hipp 2019) and *Populus* (Floate et al. 2016, Meirmans et al. 2017, Suarez-Gonzalez et al. 2018). Hybridization between sympatric tree species can increase the genetic diversity of marginal populations, lead to introgression of beneficial alleles into one of the species, and increase its adaptive potential (Hamilton and Miller 2016, Menon et al. 2020). As such, it is now considered an important evolutionary phenomenon (Whitney et al. 2010).

Even for allopatric species (i.e., spatially isolated species), the absence of physiological barriers to reproduction offers opportunities to artificially breed and translocate adapted individuals (Meirmans et al. 2010, Thompson et al. 2010, Talbot et al. 2012). There is a long history of hybridization between native and exotic tree species to meet silvicultural, ornamental or agroforestry needs (e.g., *Populus* hybrids - Park and Wilson 2007, Meirmans et al. 2010, Talbot et al. 2012; *Larix* hybrids - Meirmans et al. 2014). Such hybridization may also be used to incorporate alleles linked with pest resistance into otherwise sensitive native species (e.g., Westbrook et al. 2020).

# Appendix S2: Phenotypic plasticity meta-analysis

## Survey of plasticity studies

In December 2019, we performed searches in the Web of Science database (<https://apps.webofknowledge.com>) for six traits identified by Nicotra et al. (2010) for which phenotypic plasticity could potentially confer adaptation to climate change. We performed this search for each of our 26 study tree species. The following traits (as presented in Table S1; DRYAD link to be added upon acceptance) were searched with the following terms in the field **TOPIC**:

- **LMA:** "leaf mass per area" OR "specific leaf area” OR “specific leaf mass” OR “specific leaf weight”
- **Stomata:** "stomata size" OR "stomata density"
- **Flowering**: flower*
- **Bud burst:** "bud burst" OR "bud flush" OR "bud break"
- **Bud set:** "bud set"
- **Seeds:** "seed number" OR "seed production"

In addition, we included the following terms in each search:

**TOPIC:** plasticity OR variation OR variability *AND* **TOPIC:** (English and scientific species names)

We recorded studies that empirically and explicitly measured trait variability in natural or semi-natural habitats, and which reported data that could be used to measure trait plasticity. We did not consider studies that focused on the prediction of trait measurements across spatial and/or temporal scales (e.g., using traits in species distribution models) nor those that averaged trait variability by stand or locality. A complete list of the studies recorded is provided in Table S1.

### **Table A2.1:** Number of studies with trait data that could be used to measure phenotypic plasticity for the 26 tree species covered by our meta-analysis. LMA: leaf mass per area, Stomata: stomata size and density, Flowering: flowering time, Bud burst and Bud set: timing of such events, Seeds: number of seeds produced per reproductive event.

| **Species** | | **LMA** | **Stomata** | **Flowering** | **Bud burst** | **Bud set** | **Seeds** |
| --- | --- | --- | --- | --- | --- | --- | --- |
| **Conifers** | |  |  |  |  |  |  |
|  | *Abies balsamea* | 3 | 0 | 0 | 1 | 0 | 3 |
|  | *Larix laricina* | 1 | 0 | 1 | 2 | 0 | 1 |
|  | *Picea glauca* | 6 | 0 | 0 | 5 | 2 | 7 |
|  | *Picea mariana* | 5 | 0 | 1 | 8 | 3 | 3 |
|  | *Picea rubens* | 1 | 1 | 0 | 2 | 0 | 0 |
|  | *Pinus banksiana* | 3 | 0 | 1 | 0 | 1 | 3 |
|  | *Pinus resinosa* | 1 | 0 | 0 | 1 | 0 | 0 |
|  | *Pinus strobus* | 3 | 0 | 0 | 2 | 2 | 3 |
|  | *Thuja occidentalis* | 0 | 0 | 0 | 0 | 0 | 1 |
|  | *Tsuga canadensis* | 1 | 0 | 0 | 0 | 0 | 1 |
| **Broadleaves** | |  |  |  |  |  |  |
|  | *Acer rubrum* | 8 | 2 | 2 | 3 | 0 | 5 |
|  | *Acer saccharinum* | 1 | 0 | 0 | 0 | 0 | 0 |
|  | *Acer saccharum* | 8 | 1 | 0 | 3 | 0 | 6 |
|  | *Alnus incana subsp. rugosa* | 0 | 0 | 0 | 0 | 0 | 0 |
|  | *Betula alleghaniensis* | 4 | 0 | 1 | 2 | 0 | 1 |
|  | *Betula papyrifera* | 3 | 0 | 0 | 4 | 0 | 1 |
|  | *Fagus grandifolia* | 5 | 0 | 0 | 1 | 0 | 6 |
|  | *Fraxinus americana* | 2 | 0 | 0 | 2 | 0 | 4 |
|  | *Fraxinus nigra* | 0 | 0 | 0 | 0 | 0 | 0 |
|  | *Ostrya virginiana* | 1 | 0 | 0 | 0 | 0 | 0 |
|  | *Populus balsamifera* | 2 | 1 | 2 | 5 | 2 | 0 |
|  | *Populus grandidentata* | 0 | 0 | 0 | 1 | 0 | 1 |
|  | *Populus tremuloides* | 8 | 0 | 3 | 6 | 2 | 0 |
|  | *Prunus pensylvanica* | 1 | 0 | 0 | 0 | 0 | 0 |
|  | *Quercus rubra* | 9 | 1 | 0 | 2 | 0 | 6 |
|  | *Tilia americana* | 0 | 0 | 0 | 0 | 0 | 1 |
|  | **Number of records** | **76** | **6** | **11** | **50** | **12** | **53** |
|  | **Number of species** | **21** | **5** | **7** | **17** | **6** | **17** |

## Leaf mass per unit area (LMA) plasticity to light meta-analysis

LMA depicts the leaf dry mass per unit of leaf area and can be described as the cost of light interception on a dry mass basis (Poorter et al. 2009). Although most variation in LMA is found between species, a significant portion is present within-species and within-individuals (~24% and 11% respectively; Auger and Shipley 2013). Moreover, these proportions co-vary significantly with interspecific variability (Neyret et al. 2016), indicating that different species have different LMA plasticity (e.g., Rozendaal et al. 2006).

The environmental variable used here was light availability because it has been sufficiently studied for most tree species covered by this meta-analysis to allow comparisons between species, and because LMA is known to correlate well with local light conditions (Poorter et al. 2009). We computed the Environmentally Standardized Plasticity Index (ESPI) (Valladares et al. 2006), which corresponds to the difference in LMA between low and high light environments divided by the difference in light availability (log scale of % full sunlight) between each environment. Before calculating ESPI, values were standardized between species by dividing LMA values by the species mean LMA.

In November 2018, we performed searches in the Web of Science database for LMA plasticity to light for each of our 26 study tree species with the following terms:

**TOPIC:** (specific leaf area OR leaf mass per area OR specific leaf mass OR specific leaf weight) *AND* **TOPIC:** (variation OR plasticity) *AND* **TOPIC:** (light)

In addition, we included the following terms in each search:

*AND* **TOPIC:** (English and Latin species names)

Based on the search results, we then selected studies that corresponded to the following criteria: 1) Studies evaluated the variation in leaf mass per unit area (LMA) or its inverse, specific leaf area (SLA) in environments with different levels of light availability; 2) light availability was measured in such a way that the recorded value could be converted to % of full sunlight (i.e., without canopy interference). For example, to use values expressed as photosynthetically active radiation (PAR), an additional measurement taken under full sunlight was needed to allow data conversion.

We excluded studies that did not test the link between light availability and LMA. Therefore, we excluded many modelling experiments that predicted levels of photosynthesis based on LMA measurements. We also excluded studies that focused on hybridized species.

A total of 17 studies matched our criteria, yielding a total of 73 plasticity observations for 17 of our 26 species. These values are provided in Table S2 (DRYAD link to be added upon acceptance). SLA and LMA data were converted to the same units, i.e., g/cm^2^. In cases where data were provided only in figures, we extracted values using the *digitize* function from the *digitize* package (V.0.0.4; Poisot 2011) in the R software environment (V.3.5.0; R Core Team 2017). The percentage of light availability was log transformed (base 10) to linearize the relationship with LMA. Once all data were standardized and converted, we computed the Environmentally Standardized Plasticity Index (ESPI) from Valladares et al. (2006) using the following equation:

(High light LMA – Low light LMA)/(High Light availability(log10)- Low Light availability(log10))

To better represent interspecific differences in plasticity, we computed the average of ESPI values per species along with their standard error to evaluate the confidence in these calculations.

# Appendix S3: Population heterozygosity meta-analysis

In November 2018, we performed searches in the Web of Science database for population heterozygosity for each of our 26 study tree species with the following terms:

**TOPIC:** (genetic diversity OR heterozygosity)

In addition, we included the following terms in each search:

*AND* **TOPIC:** (English and Latin species names)

Based on the search results, we then selected studies that corresponded to the following criteria:

- The study surveyed the natural range of a tree species.
- Expected heterozygosity (*H*_e_) was calculated at the population scale (often denoted as *H*_S_) or could be derived from allele frequencies provided per population. Populations were defined as sampling locations that are separated by at least 100 km.

We avoided studies working on reclamation efforts in degraded habitats or that worked on populations with considerable presence of inter-specific hybrids. We complemented the Web of Science list of references with other studies that we were aware of. This selection yielded a total of 92 studies from which 94 *H*_s_ values were extracted. These data are available in Table S3 (DRYAD link to be added upon acceptance).

**Haplotype diversity calculation:** Although the concept of heterozygosity does not apply to haploid genomes (only one gene copy), haplotype diversity can be computed at the population level using the same equation as for *H*_e_, considering haplotypes as alleles. This diversity yields the probability that two randomly drawn alleles are different given the reported allele frequencies in the population. Because of this similarity with *H*_e_, we included haplotype diversity measures in our dataset.

**Nucleotide diversity as an alternative index of genetic diversity**

Genetic diversity can also be reported as nucleotide diversity (π), the average proportion of nucleotides that vary in a population (Leffler et al. 2012, Ai et al. 2014). This metric is based on sequenced DNA and accounts for allelic richness and abundance, but also the magnitude of the genetic distance between existing alleles (Nei and Li 1979). Sequencing technologies have only become more accessible in the last decade and relatively few studies (e.g., Pavy et al. 2012, Lu et al. 2016) present the data that would be required to measure nucleotide diversity for the set of tree species covered in our meta-analysis.

# Appendix S4: NVSGD building, Mating system, and F_IS_

### **Table A4.1:** Characteristics and metrics considered in the quantification of genetic exchange within populations (NVSGD).

| Characteristic | | Metric | Definition/rationale | Type of variable/Units | Relationship with genetic exchange | Reference |
| --- | --- | --- | --- | --- | --- | --- |
| Fecundity (F) | | Age to reproductive maturity, Seed production, Seed viability, Frequency of good seed crops | Viable seed production over a 40 years period. Seed production * seed viability * frequency of good seed crops over a 40 years period (minus the time to reach reproductive maturity). | Continuous: units are million seeds ha^-1^ per 40 yrs period. | ↗ | (Boisvert-Marsh et al. 2020) |
| Extent of genetic mixing (GM) | | Mating system, Pollination vector | The proportion of individuals originating from the mixing of different genetic sources.  GM was estimated by multiplying the scores attributed to mating system and pollination vector. | Factors with attributed scores  *Mating system*:  outcrossing = 1, outcrossing to mixed = 0.75, mixed = 0.5, mixed to selfing = 0.3 *Pollination vector*: wind=1, insects =0.5, both = 0.75 | ↗ | (Hamrick and Godt 1996, Hamrick 2004) |
| Dispersal ability (DA) | | Seed weight, Seed dispersal vector, Dispersal distance | An indicator of seed dispersal distance and of the level of longer-term genetic mixing within a population. | Continuous: Varies from 10 (low DA) to 50 (high DA). Converted to vary between 0.2 and 1 | ↗ | (Boisvert-Marsh et al. 2020) |
|  | NVSGD=F*GM*DA (in million of seeds ha^-1^ per 40 year period)  Grouped into 5 classes: very low (<1), low (1-20), intermediate (20-100), high (100-1000) and very high (>1000) | | | | | |

## Mating system

Outcrossing rates (reported as t(m)) were obtained from the TOPIC database (Aubin et al. 2020) and from literature searches. Four types of mating systems were defined based on this rate: outcrossing (0.8-1), outcrossing to mixed (0.6-0.8), mixed (0.4-0.6) and mixed to selfing (0.2-0.4). No species was reported as selfing (t(m)<0.2). In some cases, no t(m) estimates could be found so we used proxies that could inform species’ mating system and the probability of self-fertilization (e.g., dioecy). All new values obtained from literature searches have been integrated to the TOPIC database (Aubin et al. 2020) and are now accessible upon request.

# Appendix S5: Meta-analysis of population differentiation

## Data collection

We obtained F_ST_ values, from the studies identified in the *H*_S_ meta-analysis and with other studies we were already aware of. In addition to this list, we performed searches in the Web of Science database for population differentiation for each of our 26 study tree species with the following terms:

**TOPIC:** (genetic differentiation OR population differentiation)

In addition, we included the following terms in each search:

*AND* **TOPIC:** (English and Latin species names)

F_ST_ is usually reported in phylogeographic studies and was compiled as is. When multiple values were reported for different sub-regions, this metric was averaged to yield one estimate per species per study.

**
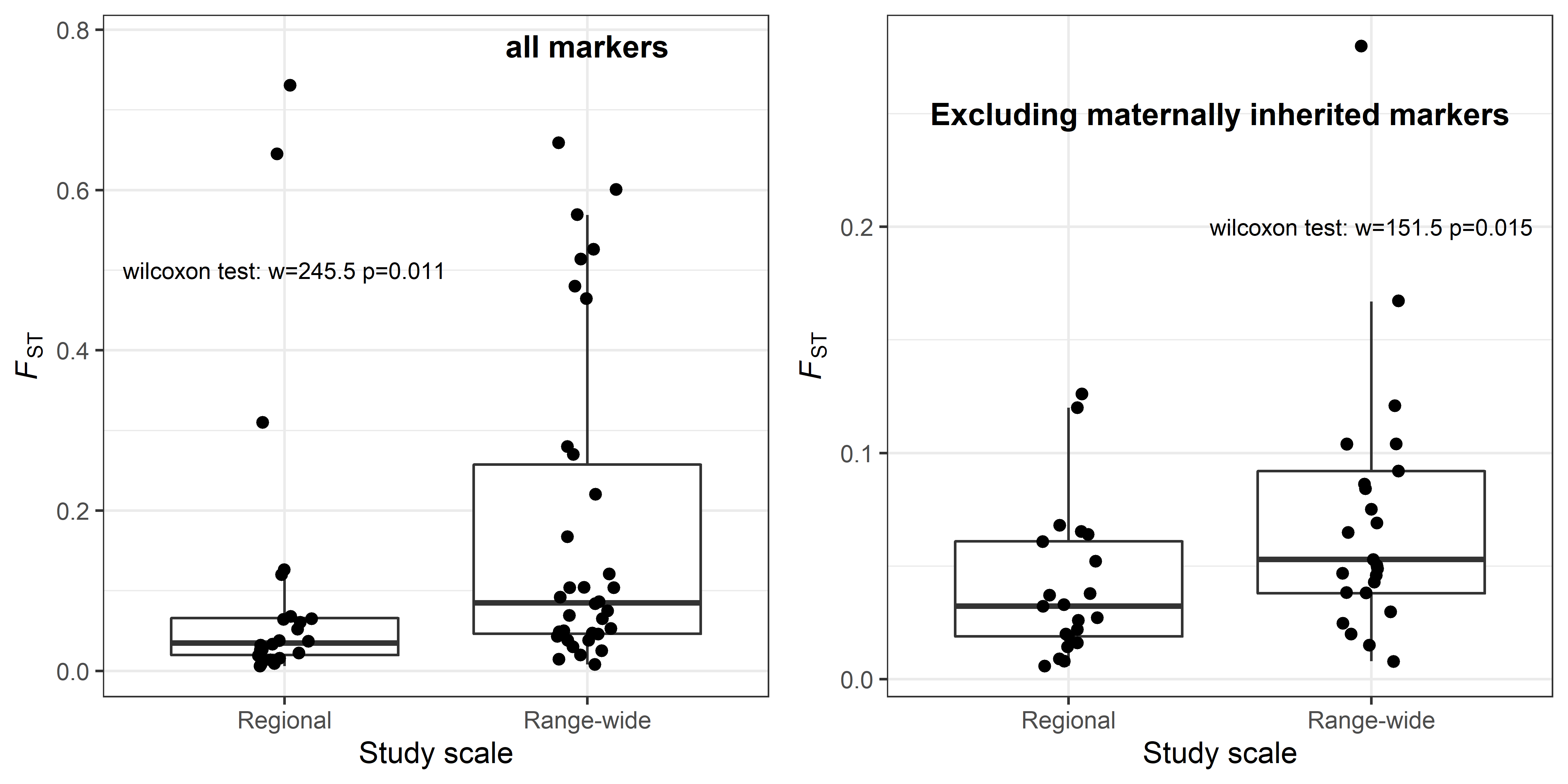
**

### **Figure A5.1:** Influence of study scale on population differentiation values (F_ST_). The

### mid-line of the boxes represents the median, the upper and lower limits of the boxes show the first and third quartiles. The lower whiskers extend to the lowest value, whereas the upper whiskers extend to 1.5 times the interquartile range (distance between first and third quartiles). Left panel: all markers included. Right panel: only the markers retained in our study are included: allozymes, RAPD, nuclear SNP, nuclear SSR, and paternal cpDNA (Figure 5 in the main text).

# References across all appendices

Agarwal, M., N. Shrivastava, and H. Padh. 2008. Advances in molecular marker techniques and their applications in plant sciences. Plant Cell Reports 27:617–631.

Ai, B., M. Kang, and H. Huang. 2014. Assessment of genetic diversity in seed plants based on a uniform π criterion. Molecules 19:20113–20127.

Aitken, S. N., and J. B. Bemmels. 2016. Time to get moving: assisted gene flow of forest trees. Evolutionary Applications 9:271–290.

Aitken, S. N., S. Yeaman, J. A. Holliday, T. Wang, and S. Curtis-McLane. 2008. Adaptation, migration or extirpation: climate change outcomes for tree populations: Climate change outcomes for tree populations. Evolutionary Applications 1:95–111.

Alberto, F. J., S. N. Aitken, R. Alia, S. C. Gonzalez-Martinez, H. Hanninen, A. Kremer, F. Lefevre, T. Lenormand, S. Yeaman, R. Whetten, and O. Savolainen. 2013. Potential for evolutionary responses to climate change evidence from tree populations. Global Change Biology 19:1645–1661.

Alfaro, R. I., B. Fady, G. G. Vendramin, I. K. Dawson, R. A. Fleming, C. Sáenz-Romero, R. A. Lindig-Cisneros, T. Murdock, B. Vinceti, C. M. Navarro, T. Skrøppa, G. Baldinelli, Y. A. El-Kassaby, and J. Loo. 2014. The role of forest genetic resources in responding to biotic and abiotic factors in the context of anthropogenic climate change. Forest Ecology and Management 333:76–87.

Allendorf, F. W. 2017. Genetics and the conservation of natural populations: allozymes to genomes. Molecular Ecology 26:420–430.

Arnold, M. L., and K. Kunte. 2017. Adaptive Genetic Exchange: A Tangled History of Admixture and Evolutionary Innovation. Trends in Ecology & Evolution 32:601–611.

Arnold, P. A., L. E. B. Kruuk, and A. B. Nicotra. 2019. How to analyse plant phenotypic plasticity in response to a changing climate. New Phytologist 222:1235–1241.

Aubin, I., F. Cardou, L. Boisvert‐Marsh, E. Garnier, M. Strukelj, and A. D. Munson. 2020. Managing data locally to answer questions globally: the role of collaborative science in ecology. Journal of Vegetation Science 31:509–517.

Aubin, I., A. D. Munson, F. Cardou, P. J. Burton, N. Isabel, J. H. Pedlar, A. Paquette, A. R. Taylor, S. Delagrange, H. Kebli, C. Messier, B. Shipley, F. Valladares, J. Kattge, L. Boisvert-Marsh, and D. McKenney. 2016. Traits to stay, traits to move: a review of functional traits to assess sensitivity and adaptive capacity of temperate and boreal trees to climate change. Environmental Reviews 24:164–186.

Auger, S., and B. Shipley. 2013. Inter-specific and intra-specific trait variation along short environmental gradients in an old-growth temperate forest. Journal of Vegetation Science 24:419–428.

Boisvert-Marsh, L., S. Royer-Tardif, P. Nolet, F. Doyon, and I. Aubin. 2020. Using a Trait-Based Approach to Compare Tree Species Sensitivity to Climate Change Stressors in Eastern Canada and Inform Adaptation Practices. Forests 11:989.

Bradshaw, A. D. 1965. Evolutionary Significance of Phenotypic Plasticity in Plants. Pages 115–155 *in* E. W. Caspari and J. M. Thoday, editors. Advances in Genetics. Academic Press.

Bussotti, F., M. Pollastrini, V. Holland, and W. Brüggemann. 2015. Functional traits and adaptive capacity of European forests to climate change. Environmental and Experimental Botany 111:91–113.

Charlesworth, D., and B. Charlesworth. 1987. Inbreeding Depression and Its Evolutionary Consequences. Annual Review of Ecology and Systematics 18:237–268.

Chmura, D. J., P. D. Anderson, G. T. Howe, C. A. Harrington, J. E. Halofsky, D. L. Peterson, D. C. Shaw, and J. B. St Clair. 2011. Forest responses to climate change in the northwestern United States: Ecophysiological foundations for adaptive management. Forest Ecology and Management 261:1121–1142.

Ellstrand, N. C., R. Whitkus, and L. H. Rieseberg. 1996. Distribution of spontaneous plant hybrids. Proceedings of the National Academy of Sciences 93:5090–5093.

Fady, B., F. A. Aravanopoulos, P. Alizoti, C. Matyas, G. von Wuehlisch, M. Westergren, P. Belletti, B. Cvjetkovic, F. Ducci, G. Huber, C. T. Kelleher, A. Khaldi, M. B. D. Kharrat, H. Kraigher, K. Kramer, U. Muehlethaler, S. Peric, A. Perry, M. Rousi, H. Sbay, S. Stojnic, M. Tijardovic, I. Tsvetkov, M. C. Varela, G. G. Vendramin, and T. Zlatanov. 2016. Evolution-based approach needed for the conservation and silviculture of peripheral forest tree populations. Forest Ecology and Management 375:66–75.

Floate, K. D., J. Godbout, M. K. Lau, N. Isabel, and T. G. Whitham. 2016. Plant–herbivore interactions in a trispecific hybrid swarm of Populus: assessing support for hypotheses of hybrid bridges, evolutionary novelty and genetic similarity. New Phytologist 209:832–844.

Franks, S. J., J. J. Weber, and S. N. Aitken. 2014. Evolutionary and plastic responses to climate change in terrestrial plant populations. Evolutionary Applications 7:123–139.

Freeman, S., and J. C. Herron. 2014. Evolutionary analysis. 5th ed. Pearson Prentice Hall, Upper Saddle River, NJ.

Gallien, L., W. Thuiller, N. Fort, M. Boleda, F. J. Alberto, D. Rioux, J. Lainé, and S. Lavergne. 2016. Is There Any Evidence for Rapid, Genetically-Based, Climatic Niche Expansion in the Invasive Common Ragweed? PLOS ONE 11:e0152867.

Ghalambor, C. K., J. K. McKAY, S. P. Carroll, and D. N. Reznick. 2007. Adaptive versus non-adaptive phenotypic plasticity and the potential for contemporary adaptation in new environments. Functional Ecology 21:394–407.

Gianoli, E., and F. Valladares. 2012. Studying phenotypic plasticity: the advantages of a broad approach. Biological Journal of the Linnean Society 105:1–7.

Gienapp, P., C. Teplitsky, J. S. Alho, J. A. Mills, and J. Merilä. 2008. Climate change and evolution: disentangling environmental and genetic responses. Molecular Ecology 17:167–178.

Godbout, J., M.-C. Gros-Louis, M. Lamothe, and N. Isabel. 2020. Going with the flow: Intraspecific variation may act as a natural ally to counterbalance the impacts of global change for the riparian species Populus deltoides. Evolutionary Applications 13:176–194.

Goulet, F., and P. Bellefleur. 1986. Leaf morphology plasticity in response to light environment in deciduous tree species and its implication on forest succession. Canadian Journal of Forest Research 16:1192–1195.

Hamilton, J. A., A. R. De la Torre, and S. N. Aitken. 2014. Fine-scale environmental variation contributes to introgression in a three-species spruce hybrid complex. Tree Genetics & Genomes 11:817.

Hamilton, J. A., and J. M. Miller. 2016. Adaptive introgression as a resource for management and genetic conservation in a changing climate. Conservation Biology 30:33–41.

Hamrick, J. L. 2004. Response of forest trees to global environmental changes. Forest Ecology and Management 197:323–335.

Hamrick, J. L., and M. J. W. Godt. 1996. Effects of life history traits on genetic diversity in plant species. Phil. Trans. R. Soc. Lond. B 351:1291–1298.

Hamrick, J. L., M. J. W. Godt, and S. L. Sherman-Broyles. 1992. Factors influencing levels of genetic diversity in woody plant species. Pages 95–124 Population Genetics of Forest Trees. Springer, Dordrecht.

Hoffmann, A. A., and C. M. Sgrò. 2011. Climate change and evolutionary adaptation. Nature 470:479–485.

Hoffmann, A. A., C. M. Sgrò, and T. N. Kristensen. 2017. Revisiting Adaptive Potential, Population Size, and Conservation. Trends in Ecology & Evolution 32:506–517.

Holderegger, R., U. Kamm, and F. Gugerli. 2006. Adaptive vs. neutral genetic diversity: implications for landscape genetics. Landscape Ecology 21:797–807.

Isabel, N., J. Beaulieu, and J. Bousquet. 1995. Complete congruence between gene diversity estimates derived from genotypic data at enzyme and random amplified polymorphic DNA loci in black spruce. Proceedings of the National Academy of Sciences 92:6369–6373.

Isabel, N., J. Beaulieu, P. Thériault, and J. Bousquet. 1999. Direct evidence for biased gene diversity estimates from dominant random amplified polymorphic DNA (RAPD) fingerprints. Molecular Ecology 8:477–483.

Jost, L. 2008. GST and its relatives do not measure differentiation. Molecular Ecology 17:4015–4026.

Jump, A. S., R. Marchant, and J. Peñuelas. 2009. Environmental change and the option value of genetic diversity. Trends in Plant Science 14:51–58.

Kattge, J., G. Bönisch, S. Díaz, S. Lavorel, I. C. Prentice, P. Leadley, S. Tautenhahn, G. D. A. Werner, et al. 2020. TRY plant trait database – enhanced coverage and open access. Global Change Biology 26:119–188.

Kokko, H., A. Chaturvedi, D. Croll, M. C. Fischer, F. Guillaume, S. Karrenberg, B. Kerr, G. Rolshausen, and J. Stapley. 2017. Can Evolution Supply What Ecology Demands? Trends in Ecology & Evolution 32:187–197.

Kremer, A., H. Caron, S. Cavers, N. Colpaert, G. Gheysen, R. Gribel, M. Lemes, A. J. Lowe, R. Margis, C. Navarro, and F. Salgueiro. 2005. Monitoring genetic diversity in tropical trees with multilocus dominant markers. Heredity 95:274–280.

Kremer, A., and A. L. Hipp. 2019. Oaks: an evolutionary success story. New Phytologist n/a.

Kremer, A., B. M. Potts, and S. Delzon. 2014. Genetic divergence in forest trees: understanding the consequences of climate change. Functional Ecology 28:22–36.

Lande, R. 2009. Adaptation to an extraordinary environment by evolution of phenotypic plasticity and genetic assimilation. Journal of Evolutionary Biology 22:1435–1446.

Ledig, F. T. 1986. Heterozygosity, heterosis, and fitness in outbreeding plants. Pages 77–104 Conservation Biology: The Science of Scarcity and Diversity. Soulé M.E. Sinauer Associates.

Leffler, E. M., K. Bullaughey, D. R. Matute, W. K. Meyer, L. Ségurel, A. Venkat, P. Andolfatto, and M. Przeworski. 2012. Revisiting an Old Riddle: What Determines Genetic Diversity Levels within Species? PLOS Biology 10:e1001388.

Lei, T. T., and M. J. Lechowicz. 1998. Diverse Responses of Maple Saplings to Forest Light Regimes. Annals of Botany 82:9–19.

Lexer, C., B. Heinze, R. Alia, and L. H. Rieseberg. 2004. Hybrid zones as a tool for identifying adaptive genetic variation in outbreeding forest trees: lessons from wild annual sunflowers (Helianthus spp.). Forest Ecology and Management 197:49–64.

Lind-Riehl, J. F., and O. Gailing. 2017. Adaptive Variation and Introgression of a CONSTANS-Like Gene in North American Red Oaks. Forests 8:3.

Loveless, M. D. 1992. Isozyme variation in tropical trees: patterns of genetic organization. New Forests 6:67–94.

Lowe, A. J., M. F. Breed, H. Caron, N. Colpaert, C. Dick, B. Finegan, M. Gardner, G. Gheysen, R. Gribel, J. B. C. Harris, A. Kremer, M. R. Lemes, R. Margis, C. M. Navarro, F. Salgueiro, H. M. Villalobos-Barrantes, and S. Cavers. 2018. Standardized genetic diversity-life history correlates for improved genetic resource management of Neotropical trees. Diversity and Distributions 24:730–741.

Lu, P., W. C. Parker, S. J. Colombo, and R. Man. 2016. Restructuring tree provenance test data to conform to reciprocal transplant experiments for detecting local adaptation. Journal of Applied Ecology 53:1088–1097.

Lustenhouwer, N., R. A. Wilschut, J. L. Williams, W. H. van der Putten, and J. M. Levine. 2018. Rapid evolution of phenology during range expansion with recent climate change. Global Change Biology 24:e534–e544.

MacLachlan, I. R., T. Wang, A. Hamann, P. Smets, and S. N. Aitken. 2017. Selective breeding of lodgepole pine increases growth and maintains climatic adaptation. Forest Ecology and Management 391:404–416.

Mahony, C. R., I. R. MacLachlan, B. M. Lind, J. B. Yoder, T. Wang, and S. N. Aitken. 2020. Evaluating genomic data for management of local adaptation in a changing climate: A lodgepole pine case study. Evolutionary Applications 13:116–131.

Meirmans, P. G., J. Godbout, M. Lamothe, S. L. Thompson, and N. Isabel. 2017. History rather than hybridization determines population structure and adaptation in Populus balsamifera. Journal of Evolutionary Biology 30:2044–2058.

Meirmans, P. G., M.-C. Gros-Louis, M. Lamothe, M. Perron, J. Bousquet, and N. Isabel. 2014. Rates of spontaneous hybridization and hybrid recruitment in co-existing exotic and native mature larch populations. Tree Genetics & Genomes 10:965–975.

Meirmans, P. G., M. Lamothe, M.-C. Gros‐Louis, D. Khasa, P. Périnet, J. Bousquet, and N. Isabel. 2010. Complex patterns of hybridization between exotic and native North American poplar species. American Journal of Botany 97:1688–1697.

Menon, M., E. Landguth, A. Leal‐Saenz, J. C. Bagley, A. W. Schoettle, C. Wehenkel, L. Flores‐Renteria, S. A. Cushman, K. M. Waring, and A. J. Eckert. 2020. Tracing the footprints of a moving hybrid zone under a demographic history of speciation with gene flow. Evolutionary Applications 13:195–209.

Mondini, L., A. Noorani, and M. A. Pagnotta. 2009. Assessing Plant Genetic Diversity by Molecular Tools. Diversity 1:19–35.

Nei, M., and W. H. Li. 1979. Mathematical model for studying genetic variation in terms of restriction endonucleases. Proceedings of the National Academy of Sciences 76:5269–5273.

Neyret, M., L. P. Bentley, I. Oliveras, B. S. Marimon, B. H. Marimon-Junior, E. Almeida de Oliveira, F. Barbosa Passos, R. Castro Ccoscco, J. dos Santos, S. Matias Reis, P. S. Morandi, G. Rayme Paucar, A. Robles Cáceres, Y. Valdez Tejeira, Y. Yllanes Choque, N. Salinas, A. Shenkin, G. P. Asner, S. Díaz, B. J. Enquist, and Y. Malhi. 2016. Examining variation in the leaf mass per area of dominant species across two contrasting tropical gradients in light of community assembly. Ecology and Evolution 6:5674–5689.

Nicotra, A. B., O. K. Atkin, S. P. Bonser, A. M. Davidson, E. J. Finnegan, U. Mathesius, P. Poot, M. D. Purugganan, C. L. Richards, F. Valladares, and M. van Kleunen. 2010. Plant phenotypic plasticity in a changing climate. Trends in Plant Science 15:684–692.

Ozinga, W. A., R. M. Bekker, J. H. J. Schaminée, and J. M. V. Groenendael. 2004. Dispersal potential in plant communities depends on environmental conditions. Journal of Ecology 92:767–777.

Paaby, A. B., and M. V. Rockman. 2014. Cryptic genetic variation: evolution’s hidden substrate. Nature Reviews Genetics 15:247–258.

Palacio‐López, K., and E. Gianoli. 2011. Invasive plants do not display greater phenotypic plasticity than their native or non-invasive counterparts: a meta-analysis. Oikos 120:1393–1401.

Paquette, A., A. Bouchard, and A. Cogliastro. 2007. Morphological plasticity in seedlings of three deciduous species under shelterwood under-planting management does not correspond to shade tolerance ranks. Forest Ecology and Management 241:278–287.

Paquette, A., B. Fontaine, F. Berninger, K. Dubois, M. J. Lechowicz, C. Messier, J. M. Posada, F. Valladares, and J. Brisson. 2012. Norway maple displays greater seasonal growth and phenotypic plasticity to light than native sugar maple. Tree Physiology 32:1339–1347.

Park, A., and E. R. Wilson. 2007. Beautiful Plantations: can intensive silviculture help Canada to fulfill ecological and timber production objectives? The Forestry Chronicle 83:825–839.

Pavy, N., M.-C. Namroud, F. Gagnon, N. Isabel, and J. Bousquet. 2012. The heterogeneous levels of linkage disequilibrium in white spruce genes and comparative analysis with other conifers. Heredity 108:273–284.

Perron, M., and J. Bousquet. 1997. Natural hybridization between black spruce and red spruce. Molecular Ecology 6:725–734.

Peterson, M. L., and K. M. Kay. 2015. Mating system plasticity promotes persistence and adaptation of colonizing populations of hermaphroditic angiosperms. American Naturalist 185:28–43.

Petit, R. J., J. Duminil, S. Fineschi, A. Hampe, D. Salvini, and G. G. Vendramin. 2005. INVITED REVIEW: Comparative organization of chloroplast, mitochondrial and nuclear diversity in plant populations. Molecular Ecology 14:689–701.

Poisot, T. 2011. The digitize Package: Extracting Numerical Data from Scatterplots. The R Journal 3:25–26.

Poorter, H., Ü. Niinemets, L. Poorter, I. J. Wright, and R. Villar. 2009. Causes and consequences of variation in leaf mass per area (LMA): a meta-analysis. New Phytologist 182:565–588.

R Core Team. 2017. R: A language and environment for statistical computing. R Foundation for Statistical Computing, Vienna, Austria.

Rice, W. R. 2002. Experimental tests of the adaptive significance of sexual recombination. Nature Reviews Genetics 3:241–251.

Rozendaal, D. M. A., V. H. Hurtado, and L. Poorter. 2006. Plasticity in leaf traits of 38 tropical tree species in response to light; relationships with light demand and adult stature. FUNCTIONAL ECOLOGY 20:207–216.

Rweyongeza, D. M., N. K. Dhir, L. K. Barnhardt, C. Hansen, and R.-C. Yang. 2007. Population differentiation of the lodgepole pine (Pinus contorta) and jack pine (Pinus banksiana) complex in Alberta: growth, survival, and responses to climate. Canadian Journal of Botany 85:545–556.

Sanford, N. L., R. A. Harrington, and J. H. Fownes. 2003. Survival and growth of native and alien woody seedlings in open and understory environments. Forest Ecology and Management 183:377–385.

Savolainen, O., T. Pyhäjärvi, and T. Knürr. 2007. Gene Flow and Local Adaptation in Trees. Annual Review of Ecology, Evolution, and Systematics 38:595–619.

Schlichting, C. D. 2008. Hidden Reaction Norms, Cryptic Genetic Variation, and Evolvability. Annals of the New York Academy of Sciences 1133:187–203.

St Clair, B. J., and G. T. Howe. 2007. Genetic maladaptation of coastal Douglas-fir seedlings to future climates. Global Change Biology 13:1441–1454.

Stapley, J., P. G. D. Feulner, S. E. Johnston, A. W. Santure, and C. M. Smadja. 2017. Recombination: the good, the bad and the variable. Philosophical Transactions of the Royal Society B: Biological Sciences 372:20170279.

Suarez-Gonzalez, A., C. A. Hefer, C. Lexer, C. J. Douglas, and Q. C. B. Cronk. 2018. Introgression from Populus balsamifera underlies adaptively significant variation and range boundaries in P.trichocarpa. New Phytologist 217:416–427.

Talbot, P., W. R. Schroeder, J. Bousquet, and N. Isabel. 2012. When exotic poplars and native Populus balsamifera L. meet on the Canadian Prairies: Spontaneous hybridization and establishment of interspecific hybrids. Forest Ecology and Management 285:142–152.

Thompson, S. L., M. Lamothe, P. G. Meirmans, P. Périnet, and N. Isabel. 2010. Repeated unidirectional introgression towards Populus balsamifera in contact zones of exotic and native poplars. Molecular Ecology 19:132–145.

Valladares, F., E. Gianoli, and J. M. Gómez. 2007. Ecological limits to plant phenotypic plasticity. New Phytologist 176:749–763.

Valladares, F., D. Sanchez‐Gomez, and M. A. Zavala. 2006. Quantitative estimation of phenotypic plasticity: bridging the gap between the evolutionary concept and its ecological applications. Journal of Ecology 94:1103–1116.

Vitti, J. J., S. R. Grossman, and P. C. Sabeti. 2013. Detecting Natural Selection in Genomic Data. Annual Review of Genetics 47:97–120.

Westbrook, J. W., Q. Zhang, M. K. Mandal, E. V. Jenkins, L. E. Barth, J. W. Jenkins, J. Grimwood, J. Schmutz, and J. A. Holliday. 2020. Optimizing genomic selection for blight resistance in American chestnut backcross populations: A trade-off with American chestnut ancestry implies resistance is polygenic. Evolutionary Applications 13:31–47.

Whitney, K. D., J. R. Ahern, L. G. Campbell, L. P. Albert, and M. S. King. 2010. Patterns of hybridization in plants. Perspectives in Plant Ecology, Evolution and Systematics 12:175–182.
